# Practical Approaches to Patient-Centered Care in Europe: Mixed Methods Study Developing a Conceptual Framework for Comprehensive Cancer Care Networks

**DOI:** 10.2196/59683

**Published:** 2025-07-31

**Authors:** Emily Hickmann, Peggy Richter, Hannes Schlieter, Maja Cemazar, Dorota Dudek-Godeau, Nele Grapentin, Ellen Griesshammer, Marjetka Jelenc, Sigita Liutkauskiene, Alain Ravaud, Xavier Troussard, Simone Wesselmann

**Affiliations:** 1 Technische Universität Dresden Faculty of Business and Economics Research Group Digital Health Dresden Germany; 2 Institute of Oncology Ljubljana Ljubljana Slovenia; 3 National Institute of Public Health National Institute of Hygiene National Research Institute Warsaw Poland; 4 German Cancer Society Berlin Germany; 5 National Institute of Public Health Research Group Raziskovalna skupina NIJZ Ljubljana Slovenia; 6 Conservative Oncology Department of Hospital of Lithuanian University of Health Sciences Kauno klinikos Oncology Institute of Lithuanian University of Health Sciences Oncology and Hematology Department Kaunas Lithuania; 7 Bordeaux University Hospital Bordeaux France; 8 University and Hospital Center Caen Normandie Caen Cedex France; 9 Deutsche Gesellschaft für Allgemein- und Viszeralchirurgie Berlin Germany

**Keywords:** comprehensive cancer care networks, oncology, patient centeredness, patient empowerment, patient engagement

## Abstract

**Background:**

In contemporary health care, patient-centered care has emerged as a pivotal paradigm shift that redefines the traditional physician-centric model. Particularly in the context of cancer care, marked by its intricate nature and emotional impact, there is a pressing requirement to rethink how health care is delivered. In this context, comprehensive cancer care networks (CCCNs) provide a new means of structuring and delivering quality cancer care, recognizing each patient’s unique preferences and needs.

**Objective:**

This study aimed to establish a consistent definition and framework for patient centeredness in CCCNs, facilitating the integration of a patient-centered approach to enhance care quality.

**Methods:**

We conducted an umbrella review focusing on generic and oncology-specific dimensions of patient centeredness to establish the definition and framework. The data were analyzed and synthesized using an inductive category development approach, which guided the derivation of dimensions for the framework. The review was complemented by a survey of 23 key stakeholders within CCCNs and a focus group with patient representatives. This process involved iterative group discussions to achieve consensus on the framework and definition.

**Results:**

The study presents a robust definition and framework of patient centeredness tailored to CCCNs, validated by an initial agreement rate of 96% among survey respondents. Patient centeredness in a CCCN is defined as a philosophy of care prioritizing the physical, emotional, and social needs and personal values of patients with cancer at every step of the patient pathway. In patient-centered CCCNs, patients are empowered and engaged in becoming active partners in health care in relation to their individual preferences and capabilities, with the goal of providing personalized, high-quality, holistic care with the best possible outcomes. The framework comprises 8 primary dimensions: empowering patients, engaging and involving patients, treating the patient as a unique person, enhancing the therapeutic relationship, enhancing a patient-centered culture, providing holistic care, recognizing and supporting the health care professional as a person, and coordinating care. Each dimension is supported by specific subdimensions and actionable patient-centered activities that facilitate practical implementation.

**Conclusions:**

The results provide a comprehensive perspective on the complex elements that compose patient-centered care within CCCNs in Europe. This contributes to a better understanding and application of patient centeredness in cancer care and possibly other contexts. The results presented in this paper promise to support cancer care networks and other health care contexts in creating a patient-centered environment where patients feel genuinely heard, valued, and actively engaged in their care decisions.

## Introduction

### Background

In the realm of contemporary health care, the concept of patient-centered care has emerged as a pivotal paradigm shift that redefines the traditional physician-centric model [1]. Patient-centered care implies empowering and engaging patients as active partners in their health care and ensuring their distinct preferences and needs are addressed [2]. The concept has been associated with multiple improved patient outcomes, encompassing higher patient satisfaction, increased patient safety, and better adherence to treatment plans while simultaneously reducing health care expenses. In addition, the focus on patient centeredness corresponds with ethical ideals such as respect for people, beneficence, and autonomy [3-5].

### Patient Centeredness in Cancer Care

Particularly in the context of cancer management, a domain characterized by its intricate nature and emotional, financial, and physical impact, there is a pressing requirement to rethink how health care is delivered by placing patients at the heart of their care [6,7]. This becomes particularly evident when considering current statistics. For instance, 30% to 55% of patients with advanced cancer have an unfulfilled information need [8]. Similarly, 10% to 24% of patients with newly diagnosed cancer report unfulfilled information needs. During the treatment phase, this unmet information need is reported by up to 97% of patients [9,10]. Further statistics show that >20% of patients do not feel sufficiently incorporated into decision-making processes, and >25% do not understand answers to their questions. These statistics are illustrative of the insufficient integration of patient centeredness in health care, thus clearly falling short in meeting the needs and preferences of patients.

### Comprehensive Cancer Care Networks

**Figure 1:**
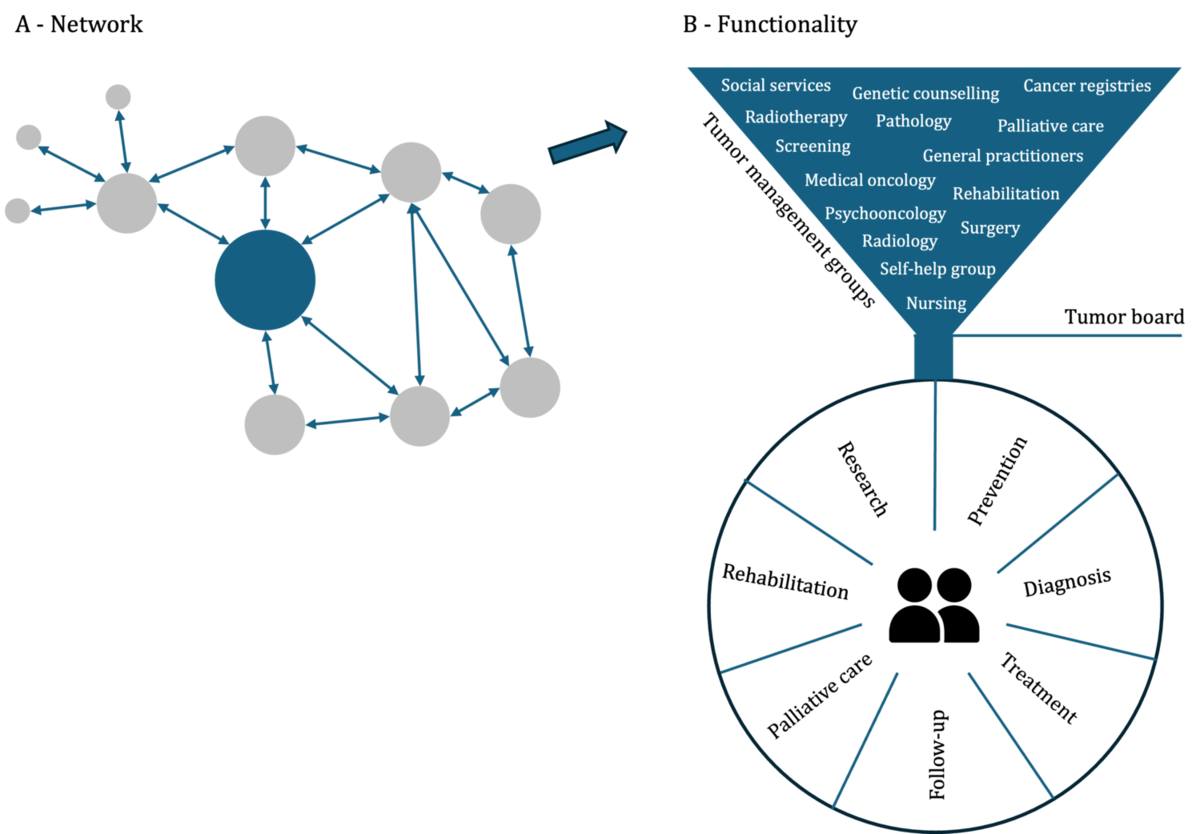
Key elements defining a comprehensive cancer care network (image based on CanCon Guide).

The evolving understanding of cancer as a complex illness has prompted a reorientation in how cancer care is structured, giving rise to comprehensive cancer care networks (CCCNs). These networks encompass various units across different institutions, covering the entire spectrum of a patient’s pathway through cancer—from diagnosis to treatment, follow-up, supportive and palliative care, and rehabilitation. These units are represented as dots in Figure 1 [11].

The tumor management groups within the CCCN are interprofessional, multidisciplinary, and tumor specific. The collaborative goal of the network is to deliver all-encompassing cancer care, focusing on enhancing outcomes and promoting consistent, high-quality care [11]. Hereby, it has been recognized that a patient-centered approach, as a cornerstone of modern, high-quality care, is indispensable [1]. Therefore, by recognizing the uniqueness of each patient’s situation, preferences, and needs, CCCNs seek to transition to a more personalized and tailored approach to cancer care [11].

### Aims

To support the shift toward a more personalized and tailored approach in cancer care, this paper aims to advance the concept of patient-centered care in CCCNs by proposing a definition, a comprehensive framework, and actionable strategies. Such a definition and framework can serve as a foundational platform for effective communication, research, and discussion. Furthermore, while existing models have defined general elements of patient-centered care [10] and identified barriers [12], there is a lack of practical guidance for CCCNs to enhance patient centeredness in their care practices.

## Methods

To develop a definition, framework, and actionable strategies for patient centeredness within CCCNs, an umbrella review, a survey with CCCN stakeholders, and 2 focus groups with patient representatives were conducted. The subsequent section will display how these methodologies were integrated and harmonized.

### Umbrella Review

The umbrella review, that is, a review compiling evidence from multiple systematic reviews [13], was performed in accordance with the framework proposed by Brocke et al [14]. The review aligns with the specifications of the PRISMA (Preferred Reporting Items for Systematic Reviews and Meta-Analyses) statement. The PRISMA checklist is provided in Multimedia Appendix 1 [15]. The objective of the review was to provide an overview of the generic and oncology-specific dimensions of patient centeredness, including any actionable patient-centered activities that may already be derived from literature, to enhance patient centeredness in practice. An umbrella review was chosen to provide a comprehensive overview of the concept of patient centeredness while still accounting for the large number of publications on the topic. The following search string was used in a PubMed query: (patient centered*[Title] OR person centered*[Title] OR patient centred*[Title] OR person centred*[Title]) AND (defin*[Title] OR concept*[Title] OR framework[Title] OR model[Title] OR element*[Title]).

Hereby, the terms patient- and person-centered care were used interchangeably. Although slight conceptual differences are described in some sources [16], they are more commonly described as synonyms [2,17]. Filters used to narrow down the search included English or German language. Furthermore, due to the nature of an umbrella review, only reviews, systematic reviews, and metareviews were included in the search. No restrictions were made concerning the date of publication.

The search intentionally included studies beyond oncology, given that general research on patient centeredness is likely applicable to oncology as well. Restricting the search would have overlooked valuable literature. To ensure relevance and validity specific to oncology, we conducted the survey and focus groups with stakeholders from the cancer field.

**Figure 2:**
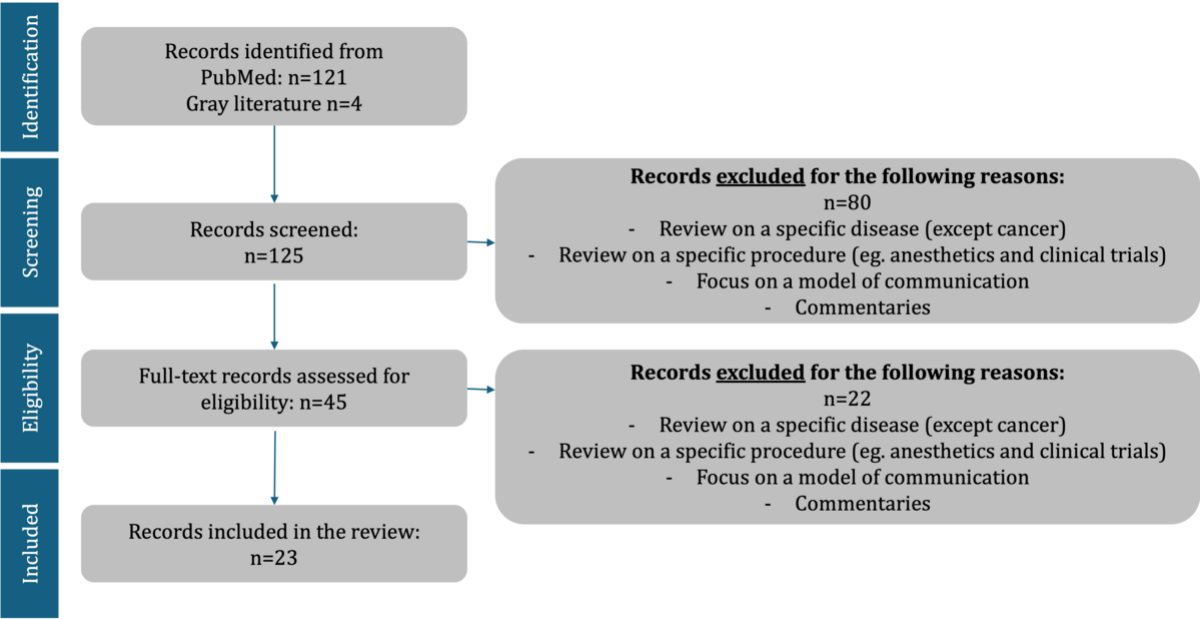
PRISMA (Preferred Reporting Items for Systematic Reviews and Meta-Analyses) flow diagram of the literature selection process.

The initial search of the databank identified 121 records. In addition, a search of gray literature using Google and Google Scholar with the term “patient centeredness” combined with those used in the PubMed query yielded 4 more sources, which were included. After a 2-stage screening process by 2 independent researchers (EH and PR), 23 records were included in the final set of publications (Figure 2 [15]).

We decided to refrain from searching further databases, as a saturation point was reached where no new dimensions of patient centeredness were identified by adding further reviews to the set of included articles. Articles were chosen for inclusion in the study if their main emphasis was on defining or investigating the concept of patient centeredness, whether in a general health care context or specifically within the field of oncology. Articles were excluded if they concentrated solely on patient centeredness related to other specific diseases outside of oncology. This approach aimed to ensure that the study remained aligned with its focus on oncology while allowing a broader exploration of patient centeredness as a concept. Furthermore, articles were included if they focused on patient centeredness in a network of health care providers. Articles that solely focused on a particular disease (except cancer), specific medical procedures (such as patient centeredness during sedation or clinical trials), communication models, or commentaries on existing articles were excluded. A list of the 23 included records, including their publication year, country, and focus, can be found in Multimedia Appendix 2 [10,17-38].

**Figure 3:**
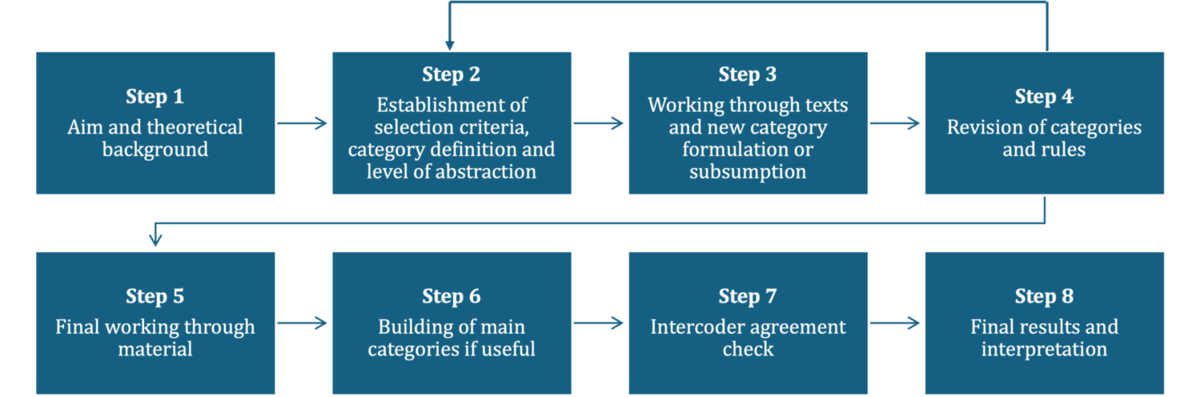
Inductive category formation approach based on the methodology by Mayring [39].

To extract information from the articles, an inductive category formation approach was used following the guidelines proposed by Mayring [39]. The steps displayed in Figure 3 [39] were performed.

The aim of the inductive category development was to identify dimensions relating to patient centeredness. To define initial categories and levels of abstraction, we used the overview of patient-centeredness dimensions by Langberg et al [18] as our foundation. This model is the most recent and comprehensive available, effectively integrating and expanding upon earlier frameworks such as that of Mead and Bower [19]. The study by Langberg et al [18] was selected not only for its up-to-date synthesis of insights but also because it remains generic enough to encompass a broad range of applications without concentrating on a specific stakeholder. Consequently, the following five dimensions proposed by Langberg et al [18] served as the starting point for our umbrella review: (1) biopsychological, (2) patient as a person, (3) sharing power and responsibility, (4) therapeutic alliance, and (5) coordinated care. As described by Mayring [39], this was a deductive element in the process, and further categories (ie, dimensions) were then inductively added if the material that was worked through could not be sorted into one of the existing categories.

The inductive category development was performed by 1 of the reviewers (EH) and constantly reviewed through group discussions with 2 further independent researchers (PR and HS). In case of dissenting views, consensus was reached through majority or through consultation of further researchers. This was an ongoing process used to revise and refine the categories and rules (step 4). Consequently, changes to the existing categories of Langberg et al [18] were made, as refined dimensions could be identified. For instance, “sharing power and responsibility” was differentiated into 2 categories: “engaging and involving patients” and “empowering patients.” After several rounds of revision, 8 categories were identified.

In this manner, the findings from the umbrella review, encompassing both generic and oncology-specific dimensions, were methodically used to establish a first draft framework of patient centeredness within CCCNs consisting of 8 main dimensions and associated subdimensions. The framework and its dimensions were also used as a basis to formulate a first approach to a definition for patient centeredness in CCCNs.

Furthermore, in each subdimension, actionable activities that could already be retrieved from the literature were recorded in a Microsoft Excel document. These patient-centered activities were collected to support the implementation of the dimensions in practice.

### Survey With Key Stakeholders in CCCNs

Subsequently, the proposed definition and framework of patient centeredness within a CCCN were transposed into a survey (Multimedia Appendix 3). The survey aimed to revise the results obtained in the umbrella review to make them more practice-oriented and applicable to the CCCN context. The reporting of the survey aligns with the CHERRIES (Checklist for Reporting Results of Internet E-Surveys) checklist for reporting results of e-surveys [40].

The survey was developed using LimeSurvey, a robust online tool that has demonstrated reliability and efficacy across various projects. To enhance the survey’s clarity, a preliminary test was conducted with 3 stakeholders, resulting in minor refinements to the wording. In addition, the platform’s inbuilt consistency check was used and did not identify any errors. The survey procedure did not require further approvals.

The survey was distributed among key stakeholders of CCCNs across various European countries. These stakeholders were identified based on their involvement in the CraNE Joint Action (European Network of Comprehensive Cancer Centers), facilitating the identification of 77 potential recipients with relevant experience in CCCNs. Key stakeholders are health care providers, such as oncologists who offer direct patient care, and managers of health care organizations responsible for the administration and coordination of services. Methodologists, such as medical guideline developers, play a crucial role as key stakeholders by setting care standards and ensuring that CCCNs operate based on the latest evidence-based practices. Representatives from national authorities and both international and national health organizations are also integral stakeholders, as they provide essential regulatory oversight and are involved in shaping policies that impact CCCNs. Including patient representatives ensures that the patient voice and perspective are central to the care process, addressing their needs and preferences effectively. In addition, researchers contribute their expertise by integrating scientific advancements and innovative approaches into cancer care. Together, these key stakeholders represent the comprehensive spectrum of expertise and experience required to deliver holistic and effective care in CCCNs.

The survey was disseminated through email, which included a hyperlink to access the online questionnaire. Participants were informed that the estimated time required for completion of the survey was approximately 20 to 30 minutes. Participants had the option to review their responses by using the back button throughout the survey. No incentives were provided for completing the survey.

Participants were asked to provide insight on the following subjects:

- Their level of agreement or disagreement with the proposed definition and framework of patient centeredness within a CCCN (5-point Likert scale).
- Recommendations for modifications to the suggested description and framework of patient centeredness within a CCCN (open text).
- What actionable and concrete patient-centered activities are currently being undertaken or could potentially be undertaken within CCCNs to enhance each of the 8 dimensions (open text).
- The extent to which patient-centered care is currently reflected in the operational practices of CCCNs concerning each specific dimension (5-point Likert scale). This topic was not confined to the particular CCCN where the participant works; instead, it encompassed their perspective on all CCCNs in general.

The survey consisted of 33 questions, organized into 10 groups, with each group displayed on a separate page. During the 3 weeks that the survey was available online, 34 potential participants visited the first survey page, and 23 complete responses were collected. This corresponds to a response rate of 30%. It was not possible for participants to make duplicate entries. Table 1 illustrates the specific stakeholder groups of a CCCN that participated in the survey.

**Table 1:** Stakeholder groups of survey respondents (N=23).

Stakeholder group	Respondents, n (%)
Health care provision	8 (35)
Cancer organization	4 (17)
Public health institute	4 (17)
Network management and certification	2 (9)
Research	2 (9)
Cancer patient organization	1 (4)
Ministry of health	1 (4)
Other	1 (4)

The results obtained from the survey, especially qualitative feedback from open-text questions, prompted adjustments to the proposed definition and framework of patient centeredness. Participants’ input informed these changes, ensuring the definition and framework remained relevant and practical for CCCNs. This iterative process underscores the importance of stakeholder engagement in refining patient-centered care models. Furthermore, the list of actionable patient-centered activities in the respective subdimensions could be expanded significantly.

### Focus Group With Patient Representatives

A focus group with 3 patient representatives, selected from the patient representative board of the medical university in Dresden, was performed. They were chosen because each representative had first-hand experience with cancer and possessed >3 years of experience serving on the board of representatives. Furthermore, these individuals served as contact people and spokespeople within the cancer care network in Dresden, providing guidance and advice to patients with cancer and fellow representatives. Thereby they offer a comprehensive understanding of the patient perspective across the entire care spectrum.

The focus group was facilitated in the following manner: in August 2023, a preparatory 1-hour kick-off meeting took place. The purpose of this session was to introduce the study’s goals, methodology, and initial findings. Following this, the patient representatives were requested to individually contemplate the preliminary results, express their thoughts, and contribute additional instances of patient-centered activities. These examples could either be ones they had personally encountered during their care or ones they would have desired within the specific context.

In October 2023, the focus group was conducted with the same 3 patient representatives. This approach should not be confused with a group interview, where the researcher directs questions, guides the discussion, or interacts individually with participants. Instead, in a focus group discussion, researchers act as moderators, facilitating group discussions among participants [41]. After a short introduction, each theme was discussed separately, and the results of the individual contemplations of each patient representative were discussed together. Commonly agreed-upon aspects were added to definitions of themes and specified in the list of patient-centered activities.

### Ethical Considerations

Participants in both the survey and focus group were informed about the study’s purpose and methodologies. By proceeding with the survey or participating in the focus group, they provided their consent to partake in the research. All collected data were aggregated to maintain participant anonymity and confidentiality. Participants of the focus group received a compensation of €90 (US $104) per hour for their involvement, acknowledging their time and contribution to the research. No incentives were provided for survey completion. The study received a retrospective ethical exemption (dated April 29, 2025) from the ethics board of the Technische Universität Dresden.

### Positionality Statement

Our research team comprises a diverse group of individuals with varied backgrounds and roles within the health care ecosystem. Some of us are researchers affiliated with universities, with expertise in health care management and business informatics. We bring an academic perspective that values evidence-based approaches and systematic analysis to understand complex health care systems. Others in our team are practitioners directly involved in cancer care or work within cancer organizations, contributing valuable on-the-ground insights from daily interactions with patients and health care systems. Our collective experiences and professional backgrounds inevitably shape our approach to this research, infusing it with both theoretical rigor and practical relevance, as we strive to enhance patient-centered care.

## Results

### Overview

**Figure 4:**
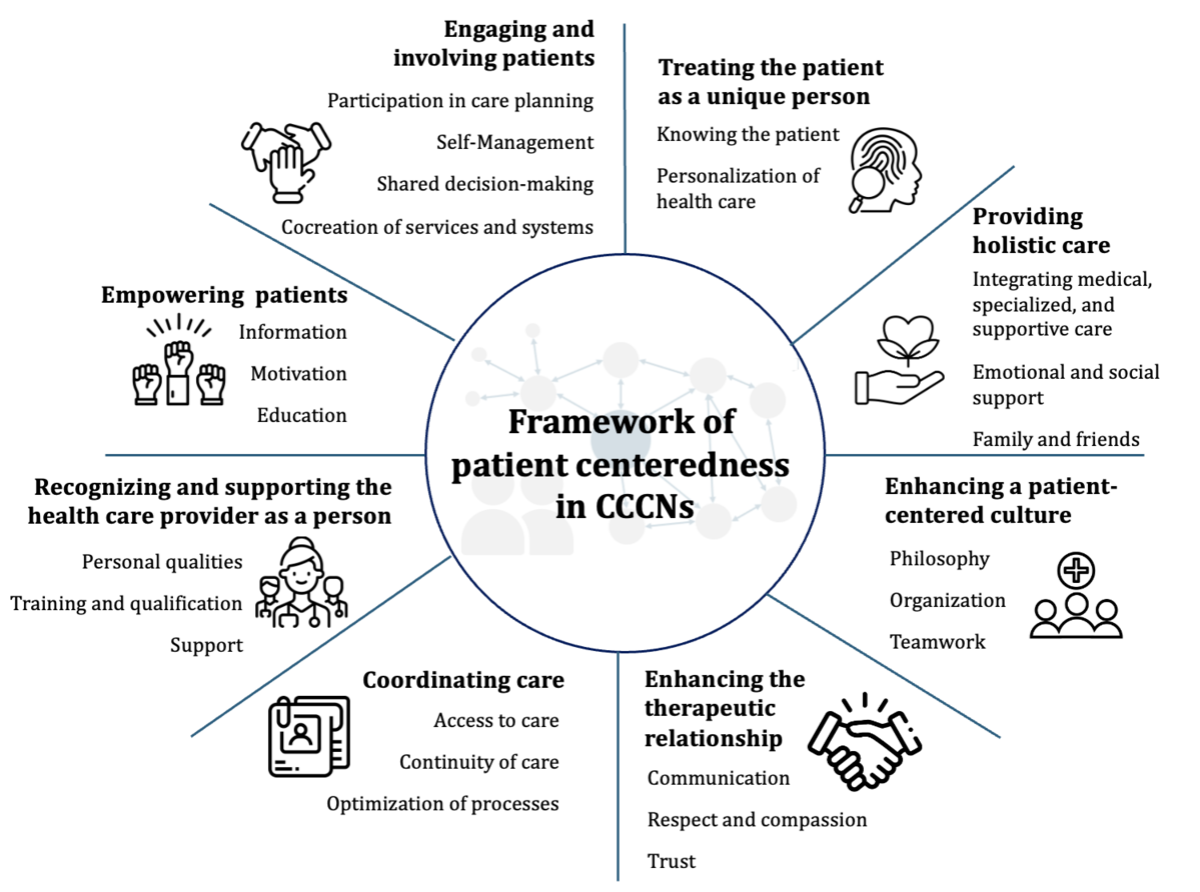
Framework of patient centeredness in comprehensive cancer care networks (CCCNs; icons created by Freepik, Kliwir art, and Samber The Labs from flaticon).

Informed by the umbrella review and survey findings, the subsequent framework (Figure 4) for patient centeredness in a CCCN was established. It consists of 8 main dimensions, each with 2 to 4 subdimensions. The framework is primarily applicable within a European context because, while the literature includes diverse perspectives from outside Europe, the survey itself was conducted exclusively with European participants. The dimensions do not have a specific order or hierarchy. Dimensions are interconnected, so a higher degree of implementation in one dimension can facilitate an easier implementation in another. For instance, if patients in a CCCN are already largely empowered, it will be easier to foster their engagement. A patient-centered culture will support the formation of a therapeutic alliance.

Out of all survey participants, 96% (22/23) of the participants either “agreed” or “strongly agreed” (on a 5-point Likert scale) with the initial framework of patient centeredness in a CCCN, which was developed based on the umbrella review results. Feedback from the survey guided revisions to the framework. Particularly, the input from the participant who did not agree with the framework prompted us to add a new subdimension, “cocreation of services and systems,” under the existing dimension of “engaging and involving patients.” This subdimension underscores the significance of involving patients in the design and improvement of health care services and systems, thereby enhancing their patient centeredness.

While the focus group with patient representatives did not result in any changes to the framework itself, adjustments were made to dimension descriptions, and additional examples of patient-centered activities were incorporated. Especially regarding patient-centered activities, patient representatives contributed unique insights and real-life examples from a patient standpoint that had not been integrated before. These modifications, prompted by the focus group discussions, are indicated in Multimedia Appendix 4 [10,17-38].

In the following subsections, the individual dimension and subdimensions of the framework for patient centeredness in CCCNs will be described, including exemplary patient-centered activities that have the potential to facilitate the practical implementation of patient centeredness in each subdimension. It is important to note that certain activities may be relevant to multiple dimensions, and there are clear synergies and overlaps between the different dimensions. Therefore, a primary allocation has been made to assign activities to specific subdimensions based on their primary focus. As the list of potential activities is very extensive, only examples will be provided in the following, while the complete list can be retrieved in Multimedia Appendix 4. In addition, the data gathered from the survey regarding the current state of implementation of patient-centered care within the operational practices of CCCNs are discussed individually for each dimension, referred to as the “status quo.”

### Empowering Patients

**Figure 5:**
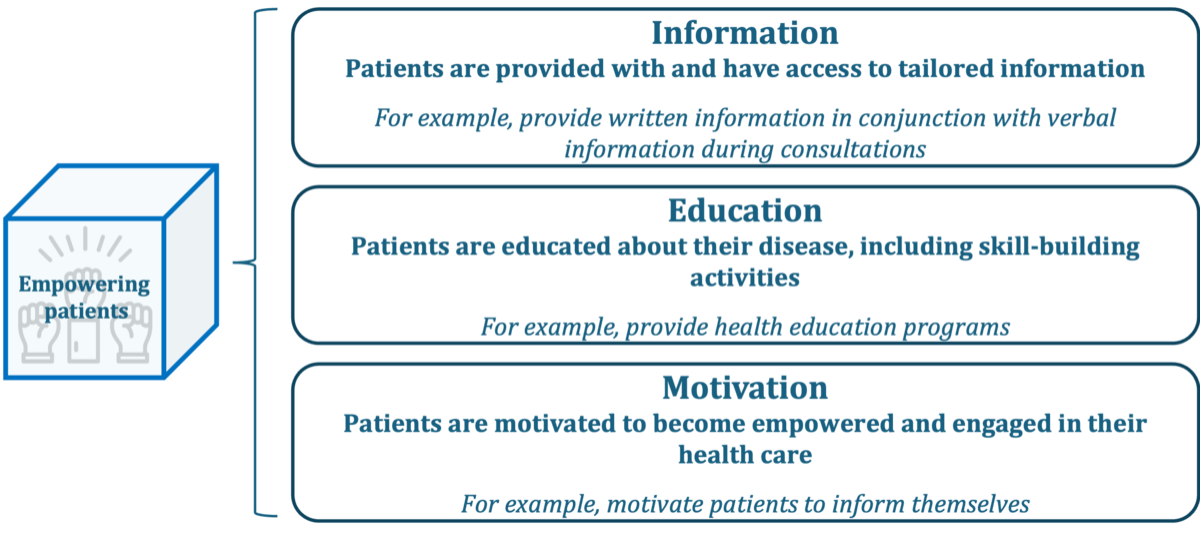
Subdimensions and examples of patient-centered activities in the dimension “empowering patients.”.

Patient empowerment refers to the process of enhancing patients’ knowledge, skills, and self-awareness while also fostering their confidence to actively participate in care [2]. Three subdimensions, displayed in Figure 5, were formed: information, education, and motivation.

Information refers to patients being provided with and having access to tailored information. Exemplary patient-centered activities found in the literature include ensuring access to care plans and health care records [20,21]; providing patients with access to audio recordings or written summaries of clinical consultations [21]; and making an information and support center available and easily accessible for staff, patients, family members, and health care professionals [22]. Examples of further activities that emerged through the survey include allocating sufficient time during consultations, establishing an informative website, and ensuring patients understand the provided information. An approach, such as the teach-back method [42], could be used for this purpose. The second subdimension focuses on patient education. Exemplary patient-centered activities found through the review process include providing health education and management programs [10,20,21,23-31], using waiting rooms and other public spaces for opportune health education [20,25], and ensuring access to patient advocacy and advisory services [10,20]. An additional activity from the survey is providing discussion groups for patients. Finally, the last subdimension emphasizes the importance of patient motivation. Hereby, health care providers can actively seek to motivate patients to engage in and take control of their health care [27]. Within the survey, respondents described the status quo of the dimension “empowering patient” as follows: 17% (4/23) to a little extent, 61% (14/23) to some extent, 13% (3/23) to a large extent, and 9% (2/23) to a considerable extent.

### Engaging and Involving Patients

**Figure 6:**
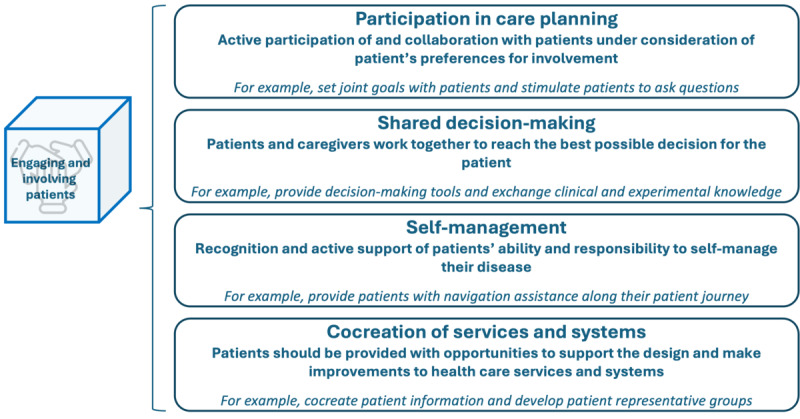
Subdimensions and examples of patient-centered activities in the “engaging and involving patients” dimension.

The dimension “engaging and involving patients” refers to the active role of patients in health care, either independently through self-management activities or in collaboration with health care professionals through shared decision-making and care planning [2]. Figure 6 displays the 4 formed subcategories: self-management, participation in care planning, shared decision-making, and cocreation of services and systems.

Self-management involves recognizing and actively supporting the patient’s ability and responsibility to manage their disease. An exemplary patient-centered activity within this subdimension is the provision of self-management tools [20,21,23,25,32,33]. An example of this, which was described in the survey, is a patient passport, which patients can use to record their cancer pathway. Further patient-centered activities in this subdimension include self-management training programs [20,21,23,25,32,33] and providing patients with navigation assistance [10,32]. Examples of additional activities outlined in the survey encompass supporting the self-management of pain and initiating specific patient engagement programs, such as self-initiated follow-ups for patients. The subsequent subdimension describes patients’ participation in care planning. Hereby, patients’ personal preferences and capabilities for participation must always be considered. Patient-centered activities described both in the literature and the survey consist of setting common goals with patients [20], encouraging patients to ask questions [23], co-designing and discussing care plans [20,27], and providing opportunities for integrating patient needs [25,31]. Within the third subdimension, shared decision-making, patients and health care professionals engage in a collaborative process aimed at reaching the optimal choices for patients’ well-being. Exemplary patient-centered activities in this subdimension encompass offering a clinical decision support system [28,32], providing decision-making tools [21,30,34,35], and exchanging clinical and experimental knowledge [36]. Another example from the survey is training patients in communication and negotiation skills. The fourth subdimension concerns the cocreation of services and systems, focusing on the organizational level. It is considered an essential aspect of patient centeredness because a service or system can arguably only achieve true patient centeredness if patients are involved in its design and implementation. Patients need to be provided with opportunities to participate in shaping and improving health care services and systems actively. The survey revealed various activities that illustrate this concept, for instance, (1) jointly developing patient information and patient pathways; (2) involving patients, their affiliated organizations, and support groups in the planning and coordination of services; and (3) engaging patients in strategic planning, guideline formulation, and policy development. Hereby, as in other dimensions, it is essential to allocate economic resources to carry out these activities. Within the survey, respondents described the status quo of the dimension “engaging and involving patients” as follows: 17% (4/23) to a little extent, 57% (13/23) to some extent, 17% (4/23) to a large extent, and 9% (2/23) to a very large extent.

### Enhancing the Therapeutic Relationship

**Figure 7:**
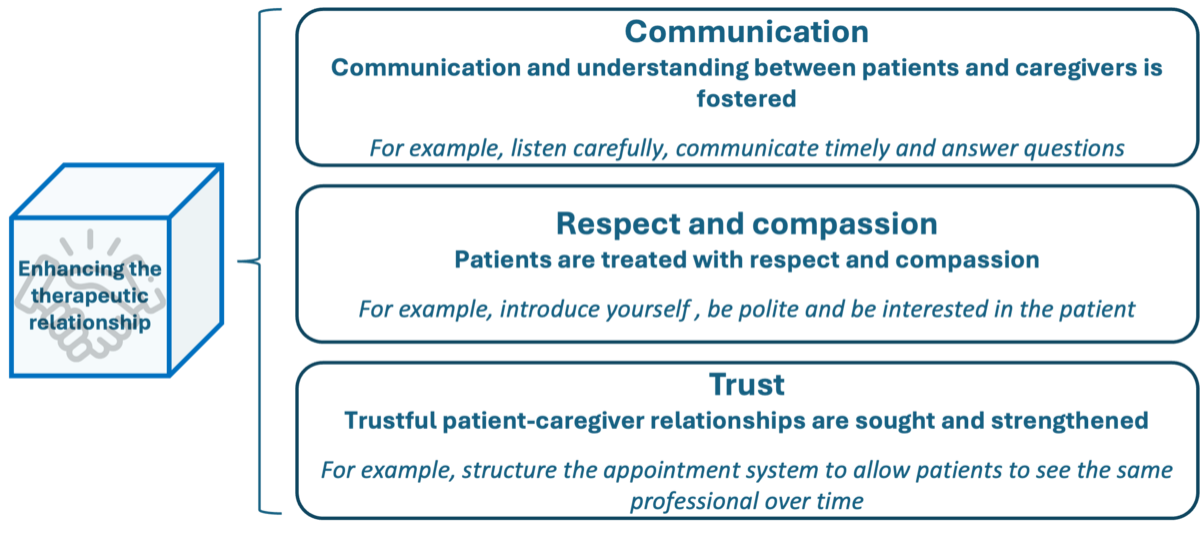
Subdimensions and examples of patient-centered activities in the “enhancing the therapeutic relationship” dimension.

The dimension “enhancing the therapeutic relationship” underscores the significant importance placed on the relationship between health care professionals and patients [18-20]. Three distinct subcategories have been identified: clinician-patient communication, respectful and compassionate care, and a trusting relationship, which are displayed in Figure 7.

Within the realm of physician-patient communication, efforts are directed toward nurturing comprehension between the parties involved. Patient-centered activities pertinent to this subdimension encompass active listening [20,23-26,32,36], engaging in timely communication [10], using nonverbal cues [23], and dedicating sufficient time [23,28]. An additional activity assembled from the survey involves implementing concise, straightforward, and effective communication and counseling protocols. To foster respectful and compassionate care, the literature highlights patient-centered activities, such as being empathic [32], warm, friendly, and approachable [10,34]; demonstrating genuine interest in the patient [23]; using polite introductions [34]; and treating the patient with dignity and respect [10,30,34,36]. The final subdimension pertains to the pursuit and reinforcement of a trusting relationship between the patient and the health care professional. Patient-centered health care professional activities identified in the literature encompass the virtues of sincerity, truthfulness, and professionalism [10,23,25,32,34,36]; safeguarding the patient’s privacy [10,23,34]; and ensuring a designated primary point of contact for the patient [23,26]. Further activities brought to light through the survey involve structuring the appointment system to facilitate continuity of care with the same health care professional over time and establishing mechanisms for direct patient-professional interaction, for example, by implementing platforms or tools that enable secure communication between patients and health care professionals, such as telemedicine or patient portals. Within the survey, respondents described the status quo of the dimension “enhancing the therapeutic relationship” as follows: 22% (5/23) to a little extent, 30% (7/23) to some extent, 39% (9/23) to a large extent, and 9% (2/23) to a very large extent.

### Treating the Patient as a Unique Person

**Figure 8:**
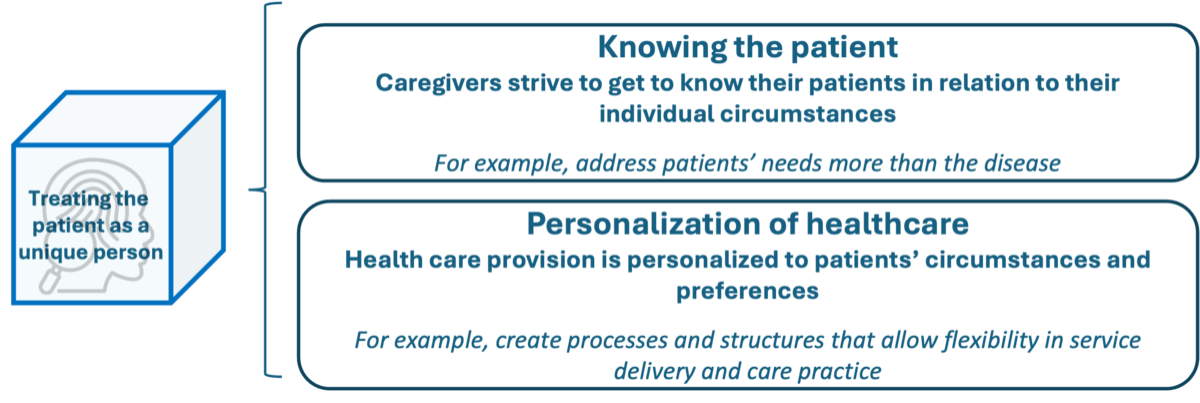
Subdimensions and examples of patient-centered activities in the dimension “treating the patient as a unique person.”.

Treating the patient as a unique person entails acknowledging the patient’s distinctive needs, preferences, values, feelings, beliefs, concerns, ideas, and expectations. An effort is made to get to know the patient, and care is personalized, respectively to the obtained knowledge [18,19,25]. Two subcategories, displayed in Figure 8, were formed: knowing the patient and personalization of health care.

Knowing the patient entails health care professionals making concerted efforts to comprehend their patients within the context of their specific situations. Noteworthy patient-centric activities within this dimension, as found in existing literature, encompass continually reassessing a patient’s objectives vis-à-vis their ongoing care plan [26], prioritizing patient needs beyond their medical condition [35], and identifying common ground based on patient preferences [30,32]. In addition, the survey yielded further examples of activities, including conducting comprehensive needs assessments to uncover patients’ priorities and giving physicians time to prepare before consultations. The second subdimension describes how health care provision is personalized to patients’ circumstances and preferences. Patient-centered activities outlined in the literature entail leveraging the knowledge of the patient as an individual to facilitate effective interactions [23,32], cultivating collaborations with community organizations [20,43], enabling home-based care possibilities [37], and creating an individualized care plan [26]. Within the survey, respondents described the status quo of the dimension “treating the patient as a unique person” as follows: 4% (1/23) to no extent, 17% (4/23) to a little extent, 35% (8/23) to some extent, 35% (8/23) to a large extent, and 9% (223) to a very large extent.

### Providing Holistic Care

**Figure 9:**
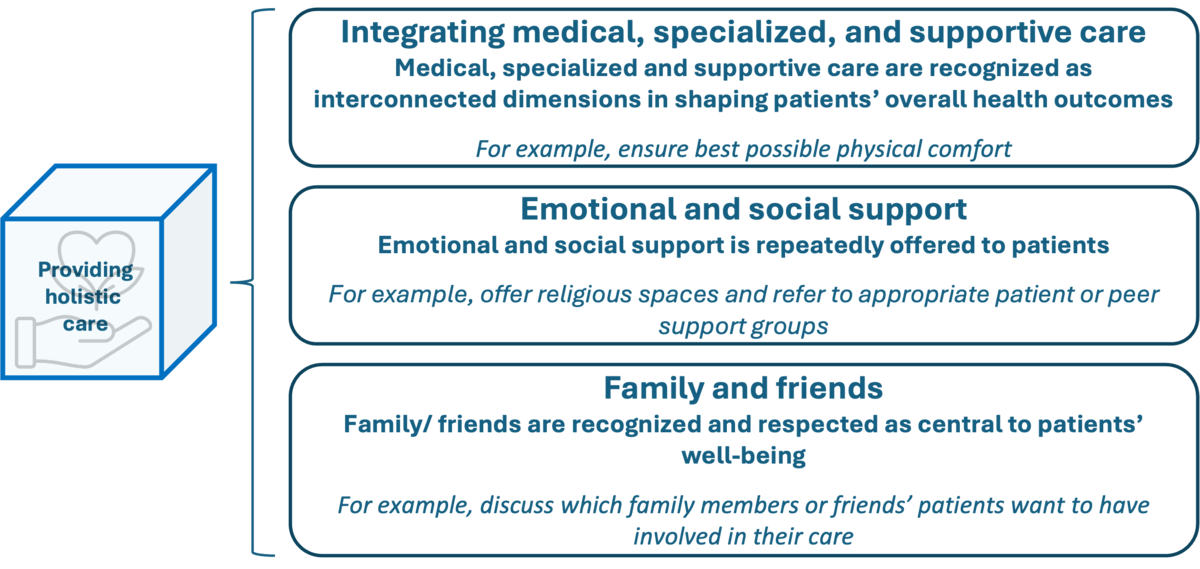
Subdimensions and examples of patient-centered activities in the dimension “providing holistic care.”.

Providing holistic care emphasizes the vital role of addressing not only the physical requirements of patients but also their emotional, social, and spiritual needs [18,19,31,38]. The dimension is further divided into 3 subdimensions, as displayed in Figure 9: integrating medical, specialized and supportive care, emotional and social support, and family and friends.

Medical, specialized, and supportive care are recognized as interconnected dimensions in shaping patients’ overall health outcomes. Hereby, specialized care plays a crucial role in tailoring interventions and treatments to specific medical conditions, ensuring precision and effectiveness in addressing the unique health care needs of individual patients. Furthermore, supportive care is recognized as an integral part of care provision. Patient-centered activities sourced from the literature encompass initiatives such as identifying patients who could benefit from early palliative care [33,37], facilitating the continuity of everyday routines and self-identity [17], ensuring optimal physical comfort [33], and specifically addressing the needs of survivors (eg, fear of recurrence) [35]. Examples of other activities mentioned in the survey include providing access to auxiliary disciplines or guidance on supportive care possibilities. The second subdimension, focused on emotional and social support, underscores the provision of emotional and social assistance. Literature highlights measures such as cultivating a supportive and accommodating environment [20,21,32,36], allocating resources for self-help programs [21], offering spaces for religious practices [20,32], and providing emotional support while being empathetically present [28,31,32]. The survey also draws attention to the importance of access to psycho-oncology services. The third subdimension, family and friends, acknowledges the central role of these individuals in patients’ well-being. Notable patient-centered activities delineated in the literature include ensuring the availability of spiritual care support for families [34], addressing the needs of family members and friends [17], and encouraging the participation of family and friends in consultations [33]. As publicized in the survey, an additional patient-centered activity involves establishing an appropriate and flexible visiting policy. Within the survey, respondents described the status quo of the dimension “providing holistic care” as follows: 9% (2/23) to no extent, 13% (3/23) to a little extent, 39% (9/23) to some extent, 35% (8/23) to a large extent, and 4% (1/23) to a very large extent.

### Recognizing and Supporting the Health Care Provider as a Person

**Figure 10:**
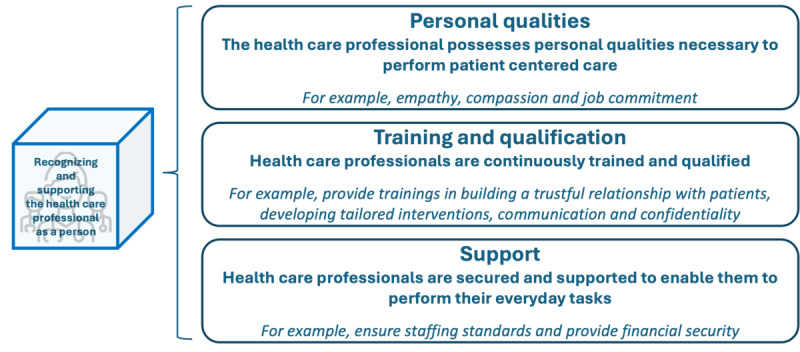
Subdimensions and examples of patient-centered activities in the dimension “recognizing and supporting the health care provider as a person.”.

“Recognizing and supporting the health care provider as a person” emphasizes the significance of acknowledging the pivotal role of health care professionals; the unique challenges they face; and the need to provide multifaceted support, including emotional, informational, and instrumental assistance [18-20]. The concept delineates 3 distinct subdimensions: personal qualities, training and qualification, and support, which are displayed in Figure 10.

In the first subdimension, the health care professional embodies personal qualities essential for patient-centered care. The literature underscores traits such as commitment to their job [28], dedication to quality [21], fostering respect and nonjudgmental behavior [25], exhibiting self-reflective behavior [23,28,31], demonstrating empathy and compassion [23,31], and adhering to principles of honesty [31]. The second subdimension emphasizes the continuous training and qualification of health care personnel. Exemplary activities found in the literature include ensuring a well-rounded skill mix [28]; promoting lifelong learning [21]; offering opportunities for continuous professional development [21,25,28,30,34]; advocating evidence-based practice [21]; and providing training in patient centeredness, effective communication, and self-care [21,22,32,34]. The third subdimension pertains to supporting health care professionals to enable them to carry out their daily tasks effectively. Activities deemed patient centered, both within the literature and gleaned from the survey, involve delivering sustained support to health care professionals throughout the care process, ensuring financial stability, providing psychological assistance, implementing incentive packages based on performance, offering recreational programs, and fostering a balance between work and personal life [21,34]. Within the survey, respondents described the status quo of the dimension “recognizing and supporting the health care professional as a person” as follows: 9% (2/23) to no extent, 35% (8/23) to a little extent, 35% (8/23) to some extent, 17% (4/23) to a large extent, and 4% (1/23) to a very large extent.

### Coordinating Care

**Figure 11:**
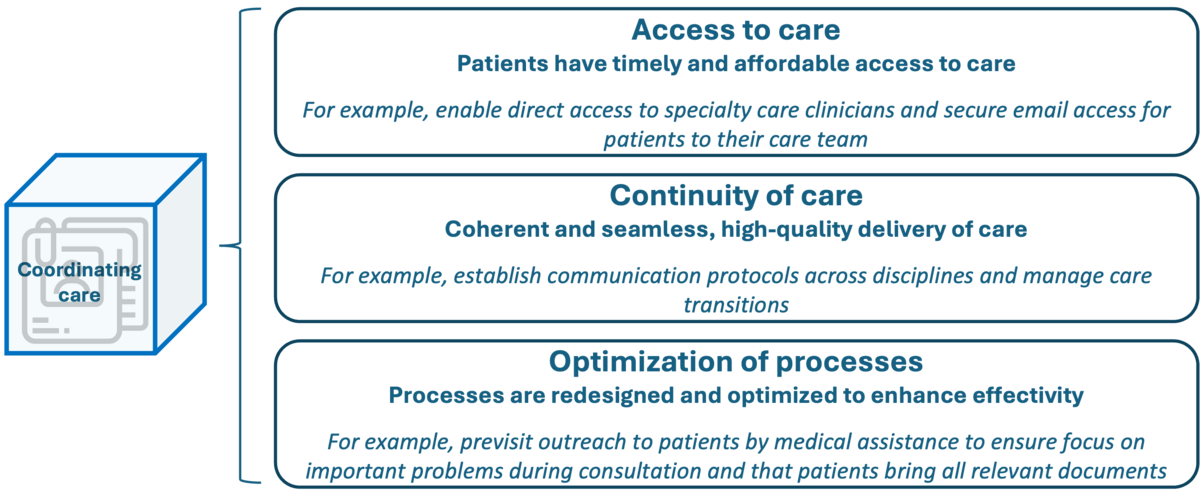
Subdimensions and examples of patient-centered activities in the dimension “coordinating care.”.

Coordinating care highlights the need to structure care processes, promote access, and ensure the continuity of care [18,20]. This concept unfolds into 3 subdimensions, as displayed in Figure 11: access to care, continuity of care, and optimization of processes.

In the access to care subdimension, the emphasis is on timely and affordable care provision. Notable patient-centered activities within this subdimension, as documented in the literature, encompass enabling secure email access between patients and clinicians [32], providing direct access to specialty care clinicians [32], implementing suitable appointment scheduling [21,32,33], and ensuring care affordability [20,32]. The survey further highlighted an additional activity: ensuring universal access to both primary and secondary care. The second subdimension, dedicated to continuity of care, revolves around delivering coherent, uninterrupted, high-quality care. Patient-centered activities highlighted in the literature include establishing interprofessional leadership teams [21], using patient pathways [32], conducting posttreatment follow-ups [10,29], and guaranteeing the seamless flow of information [20,32,37]. In addition, the survey revealed further activities, such as involving and supporting the primary family caregiver, instituting a nurse navigator, and defining wait times for diagnosis. Hereby, achieving a delicate balance between standardizing high-quality care and permitting adequate flexibility and individualization becomes essential. The third subdimension, optimization of processes, focuses on refining processes to enhance effectiveness. Patient-centered initiatives sourced from the literature encompass developing discharge and referral protocols [21], ensuring efficient workflow [30,32], minimizing clinical wait times [32], fostering continuous quality improvement [26], appointing case managers or oncology nurses with case management duties for patients with cancer [29], and implementing transparent and understandable service protocols to improve patient flow [21]. Within the survey, respondents described the status quo of the dimension “coordinating care” as follows: 13% (3/23) to a little extent, 39% (9/23) to some extent, 35% (8/23) to a large extent, and 13% (3/23) to a very large extent.

### Enhancing a Patient-Centered Culture

**Figure 12:**
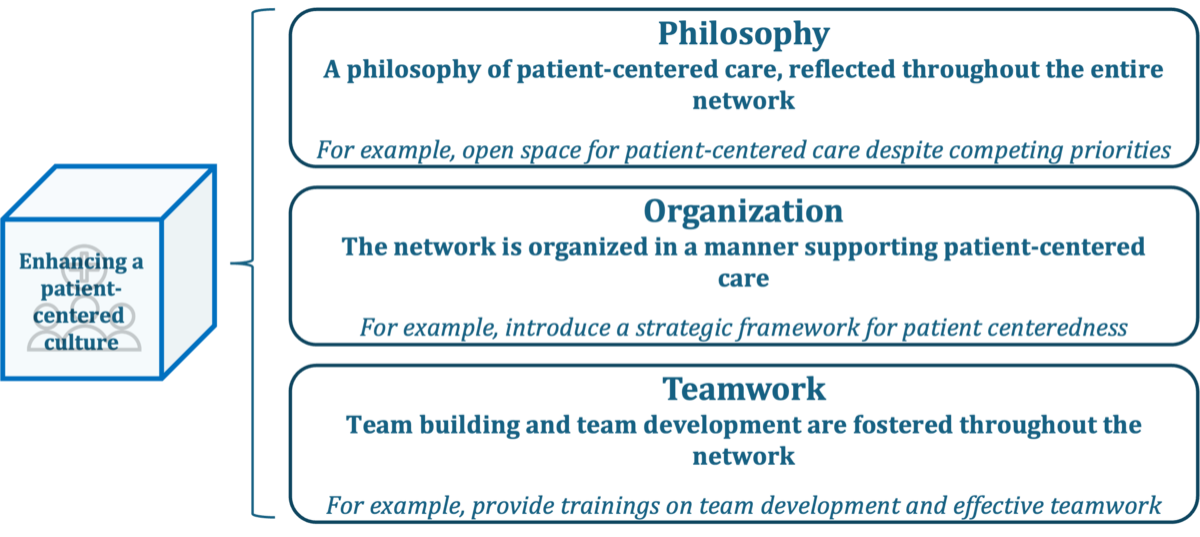
Subdimensions and examples of patient-centered activities in the dimension “enhancing a patient-centered culture.”.

Enhancing a patient-centered culture underscores the imperative of a profound cultural shift, where patient centeredness becomes deeply ingrained in the foundational philosophy and organizational structure of the health care network [20,24,25]. This dimension, displayed in Figure 12, is segmented into 3 subdimensions: philosophy, organization, and teamwork.

In the philosophy subdimension, patient-centered care permeates the entire network. Patient-centered activities documented in the literature within this subdimension encompass positioning patient-centered care as a major priority in the CCCN, fostering a culture of reflective practice [21], and embracing corporate responsibility for equality and diversity [34]. This implies that all stakeholders, for instance, medical staff and management, value the concept of patient centeredness and promote the conceptual framework. Additional patient-centered activities surfaced from the survey, including positioning patient-centered care as a primary priority within the CCCN, instilling trust and respect as core values of the CCCN, and establishing a patient charter. The second subdimension, organization, revolves around structuring the network to support patient-centered care. This encompasses patient-centered activities outlined in the literature, such as establishing services that promote a patient-centered culture [20,24,28,34]. This could, for instance, include interpretation, language, or information services. Further activities found in the literature include introducing a strategic framework for patient centeredness [28] and implementing change management aimed at anchoring patient-centered care in the everyday culture of the network [28]. Teamwork, including team building and development, is the final subdimension in the dimension “enhancing a patient-centered culture.” Exemplary patient-centered activities found in the literature include establishing and strengthening a multidisciplinary and interprofessional patient care team [21,26,29], encouraging teamwork and team building [20,28], and ensuring a strong team leadership committed to a patient-centered culture [17,24,34]. Within the survey, respondents described the status quo of the dimension “enhancing a patient-centered culture” as follows: 9% (2/23) to no extent, 13% (3/23) to a little extent, 65% (15/23) to some extent, 4% (1/23) to a large extent, and 9% (2/23) to a very large extent.

### Defining Patient Centeredness in CCCNs

Informed by the umbrella review and survey findings, the subsequent definition for patient centeredness within a CCCN was formulated: patient centeredness in a CCCN is a philosophy of care prioritizing the physical, emotional, and social needs and personal values of the patients with cancer at every step of the patient pathway. In patient-centered CCCNs, patients are empowered and engaged in becoming active partners in health care in relation to their individual preferences and capabilities with the goal of providing personalized, high-quality, holistic care with the best possible outcomes.

Out of the 23 participants who completed the survey, 22 (96%) either strongly agreed (11/23, 48%) or agreed (11/23, 48%) with the proposed definition of patient centeredness in CCCNs. The comments received from the participants supported the revision of the definition. Notably, participants highlighted that the level of patient involvement as “active” partners in care should also consider a patient’s mental and physical capabilities. Therefore, the phrase “and capabilities” was added to the definition after analyzing the results of the survey. The participant who disagreed with the definition supported the revision of the framework but did not provide additional comments specifically addressing the definition. The feedback from patient representatives during the focus group did not lead to changes in the definition.

## Discussion

### Principal Findings

This study examines the concept of patient-centered care within the context of CCCNs in Europe. In this context, the study’s findings elucidate a comprehensive definition and framework of patient centeredness. The identified framework, comprising 8 primary dimensions and 24 subdimensions, provides a structured understanding of the diverse facets that constitute patient-centered care. The combination of methods—an umbrella review, a survey of key stakeholders, and feedback from patient representatives—contributes to the robustness of the study’s findings by capturing both theoretical and a multitude of practical perspectives.

### Theoretical and Practical Contribution

The theoretical contribution of this paper lies in its support for the further conceptualization of patient centeredness within health care, particularly in the context of oncology and CCCNs. By using a robust method and delineating a comprehensive framework consisting of 8 primary dimensions and their corresponding subdimensions, this study refines and expands the understanding of patient-centered care. The framework not only encompasses the conventional theoretical elements of patient-centered care but also tailors them to suit the complex dynamics of CCCNs. The foundational principles, which could potentially apply to other medical conditions as well as singular cancer care institutions, were primarily derived from the umbrella review. However, the emphasis on cancer, particularly within the context of care delivery in CCCNs, was established by incorporating reviews specifically addressing patient centeredness in oncology, conducting surveys with key stakeholders within CCCNs, and facilitating the focus group with patients with cancer.

From a practical standpoint, the intention of this paper is to provide health care professionals, managers and coordinators of health care services, authorities, and further CCCN stakeholders with guidance to support the implementation of patient-centered care. Thus, the aim of this paper is to empower CCCNs in establishing an environment where patients feel genuinely heard, valued, and actively engaged in care. The paper offers concrete guidance and inspiration on what patient-centered activities can be put into action by CCCNs to enhance their focus on patients. The establishment of a consistent definition and framework of patient centeredness in CCCNs provides a foundation for health care institutions to prioritize patient needs and preferences, potentially leading to improved patient outcomes and experiences. By identifying specific actionable activities under each subdimension of the framework, the study provides tangible actions that CCCNs can take to implement patient-centered care effectively (complete list provided in Multimedia 4). These practical insights go beyond vague principles, empowering CCCNs and health care professionals with targeted strategies to, for instance, enhance patient engagement, promote a patient-centered culture, and establish effective therapeutic relationships.

In Multimedia Appendix 4, only the activities highlighted in light red, as well as the results obtained through the survey and the focus groups, are in direct relation to cancer care. The relevance of these activities to alternative contexts and diseases remains uncertain and necessitates further examination and empirical validation. However, the remaining actionable patient-centered activities exhibit a generic essence, suggesting their potential pertinence to diverse health care domains and institutions. This assumption lays the groundwork for their likely applicability to a range of health care entities beyond the scope of this study.

### Comparison With Prior Work

This study shares several similarities with existing conceptualizations of patient centeredness, while also introducing notable differences. As the study is rooted in a thorough analysis of existing literature and frameworks, key principles, such as the therapeutic alliance, engagement, and holistic care, are integrated [18,19,31]. To discern the differences, especially in comparison to the dimensions established by Langberg et al [18], building on the study by Mead and Bower [19], is interesting, as this was the starting point for the category formation approach used in this study. Therefore, the dimensions formulated by Langberg et al [18] are clearly reflected in the results of this study. However, a new dimension, “enhancing a patient-centered culture,” was added. Furthermore, the dimension “sharing power and responsibility” was divided into the 2 distinct concepts of patient engagement and empowerment [2]. Notably, the subdimensions provided in the framework of patient centeredness in CCCNs exhibit further differences. For instance, “providing holistic care,” which aligns with the “biopsychological” dimension by Langberg et al [18], now includes involvement of family and friends.

These differences may stem from several factors, the most obvious being the focus on cancer care in CCCNs, informed incorporation of literature based in oncology, the survey with key CCCN stakeholders, and the focus group with patient representatives in the field of oncology. Therefore, these delineations may be unique to the realm of cancer care in CCCNs. Another possibility is that the concept of patient centeredness has advanced in the last 5 years. This may be especially true, as other studies published at a later point in time (Multimedia Appendix 2) were also used as a basis for this paper.

Overall, our findings align with and extend existing theories of patient-centered care, reinforcing the notion that patient centeredness goes beyond mere patient satisfaction and, for instance, involves active patient participation, individualized care, and the recognition of the patient’s psychological and emotional state [18].

### Limitations

A limitation of this study, which warrants consideration, is the limited number of both included papers and participants. An umbrella review was chosen because of the large number of publications on the topic of patient centeredness. Only using existing reviews as a basis may warrant limitations, as it facilitates a dependence on the high quality of the performed reviews, and certain aspects of patient centeredness may have been missed. For instance, the topic of cancer survivorship is not addressed as prominently as we might have expected. Including more databases might have highlighted this topic more effectively. However, we observed a saturation of information regarding each dimension and subdimension, which likely moderated this effect. In addition, survey participants are experts in CCCNs with extensive experience either working within them or collaborating closely. Therefore, despite the limited number of participants, the quality of their responses is substantial.

It should also be considered that a patient-centered approach can vary from one patient to another. The diverse range of patient requirements, considerations, and preferences plays a role here. For instance, the level of active involvement a patient desires in their care can differ greatly. A patient-centered activity listed in Multimedia Appendix 1 may be ideal for one patient but unnecessary—or even unwelcome—for another. Moreover, the activities presented encompass a spectrum of possibilities aimed at enhancing patient-centered care. However, their suitability, feasibility, and acceptance can differ across various scenarios, necessitating further evaluation and testing. As the umbrella review focused exclusively on publications in English or German, it becomes imperative to conduct evaluations in various countries and among diverse cultural backgrounds.

### Future Research

Building on the foundation established by this study, several promising avenues for future research emerge. One potential direction is to conduct empirical investigations that assess the implementation and effectiveness of the proposed patient-centered activities within CCCNs. This could involve longitudinal studies tracking patient outcomes, experiences, and health care provider practices before and after adopting certain activities. Such research could shed light on the tangible impacts of patient-centered care on patient satisfaction, treatment adherence, and overall well-being. In addition, these studies could help confirm the study’s relevance in broader contexts, such as specific cancer care institutions or health care settings outside Europe.

In addition, exploring the contextual factors that facilitate or hinder the integration of specific patient-centered activities could provide insights into the dynamic nature of health care systems and inform strategies for successful implementation. Factors such as the competence and mindset of the medical team, economic constraints, legislation, and the interoperability or current state of digital health technologies may significantly influence the success of specific activities.

In terms of implementation support, reviews, best practices, and specific tool support would be desirable to enhance and facilitate the implementation of the individual patient-centered activities in CCCNs.

### Conclusions

This study contributes to the understanding of patient-centered care in the unique context of cancer care. By providing a comprehensive framework and definition, the study equips CCCNs, and possibly also individual cancer care institutions, with actionable patient-centered activities to foster an environment where patients are actively engaged, valued, and empowered in their cancer care pathway. As health care systems strive to prioritize patient centeredness, the insights presented here hold the potential to reshape cancer care practices and inspire broader improvements in health care delivery.

## Appendix

Multimedia Appendix 1 PRISMA (Preferred Reporting Items for Systematic Reviews and Meta-Analyses) 2020 checklist.

Multimedia Appendix 2 List of records included in the umbrella review.

Multimedia Appendix 3 Survey questions for key stakeholders in comprehensive cancer care networks.

Multimedia Appendix 4 Dimensions and possible activities of patient-centered care in comprehensive cancer care networks.

## Abbreviations

CCCN: comprehensive cancer care network
CHERRIES: Checklist for Reporting Results of Internet E-Surveys
PRISMA: Preferred Reporting Items for Systematic Reviews and Meta-Analyses

## Data Availability

The datasets generated or analyzed during this study are available from the corresponding author on reasonable request.
