# Supplementary material for: Practical Approaches to Patient-Centered Care in Europe: Mixed Methods Study Developing a Conceptual Framework for Comprehensive Cancer Care Networks
Source: JMIR Cancer. 2025 Jul 31;11:e59683. doi: 10.2196/59683 (PMC12355145; doi:10.2196/59683)
Supplement: Multimedia Appendix 2 [file cancer_v11i1e59683_app2.docx]

## Multimedia Appendix 2: List of records included in the systematic review

The systematic literature review resulted in the compilation of the definitive list of included articles, as shown in the following table. The light blue shading indicates that the source is cancer specific. Countries with an asterisk (*) pertain to the native country of the first author, as there was no mention of the publication's country of origin.

| **Authors/ Organization** | **Year** | **Country** | **Focus** |
| --- | --- | --- | --- |
| American Geriatrics Society Expert Panel on Person-Centered Care [1] | 2015 | America | Definition of person-centered care and essential elements |
| Brickley B, Sladdin I, Williams LT, Morgan M, Ross A, Trigger K, et al. [2] | 2020 | Australia* | Model of patient-centered care for general practitioners |
| British Columbia Ministry of Health [3] | 2015 | Canada | Patient-centered care in the health system and strategic plan |
| Byrne A-L, Baldwin A, Harvey C. [4] | 2020 | Singapore | Definition of patient-centered care relevant to nursing practice |
| Castro EM, Van Regenmortel T, Vanhaecht K, Sermeus W, Van Hecke A.[5] | 2016 | Belgium* | Concept analysis on the terms: patient empowerment, patient participation, and patient-centeredness |
| Evans JM, Matheson G, Buchman S, MacKinnon M, Meertens E, Ross J, et al. [6] | 2015 | Canada* | Integrating cancer care beyond the hospital and across the cancer pathway |
| Giusti A, Nkhoma K, Petrus R, Petersen I, Gwyther L, Farrant L, et al. [7] | 2020 | UK* | Conceptualization of person-centered care for serious illness |
| Greene SM, Tuzzio L, Cherkin D. [8] | 2012 | USA* | Framework to establish patient-centered care |
| Holmström I, Röing M. [9] | 2010 | Sweden* | Relation between patient-centeredness and patient empowerment |
| Langberg EM, Dyhr L, Davidsen AS.[10] | 2019 | Denmark* | Definition/ development of the concept of patient-centeredness |
| Louw JM, Marcus TS, Hugo JFM. [11] | 2017 | South Africa | Essential elements, ethical principles, and the practical application of person-centered practice |
| Mead N, Bower P.[12] | 2000 | UK* | A conceptual framework of patient-centeredness, measuring the process and outcomes of patient-centered care |
| Mitchell K-AR, Brassil KJ, Rodriguez SA, Tsai E, Fujimoto K, Krause KJ, et al. [13] | 2020 | USA* | Operationalizing patient-centered care: constructs of cancer patients’ values, needs and preferences |
| Oberst S. Organization of European Cancer Institutes [14] | 2021 | Belgium* | Core quality standards for cancer |
| Olson AW, Stratton TP, Isetts BJ, Vaidyanathan R, C Van Hooser J, Schommer JC. [15] | 2021 | USA* | Patient-centeredness conceptualization from different healthcare professional groups |
| Ouwens M, Hermens R, Hulscher M, Vonk-Okhuijsen S, Tjan-Heijnen V, Termeer R, et al. [16] | 2010 | Netherlands* | Indicators for patient-centered cancer care |
| Pel E, Engelberts I, Schermer M. [17] | 2021 | Netherlands* | Patient-centered care for breast cancer patients |
| Robinson JH, Callister LC, Berry JA, Dearing KA. [18] | 2008 | America* | Definition and application of person-centered care |
| Santana MJ, Manalili K, Jolley RJ, Zelinsky S, Quan H, Lu M. [19] | 2017 | Canada* | Conceptual framework of person-centered care |
| Scholl I, Zill JM, Härter M, Dirmaier J. [20] | 2014 | USA | Dimension and integrative model of patient-centeredness |
| Symingtom D. National Health Service [21] | 2017 | Scotland* | Person-centered health and care framework |
| World Health Organization [22] | 2007 | Switzerland* | Policy framework of people-centered health care |
| Zucca A, Sanson-Fisher R, Waller A, Carey M.[23] | 2014 | Germany | Conceptualization and measurement of patient-centered care |

1. (30) American Geriatrics Society Expert Panel on Person-Centered Care. Person-Centered Care: A Definition and Essential Elements. J Am Geriatr Soc. 2016;64:15–8.

2. (27) Brickley B, Sladdin I, Williams LT, Morgan M, Ross A, Trigger K, et al. A new model of patient-centred care for general practitioners: results of an integrative review. Fam Pract. 2020;37:154–72.

3. (28) The British Columbia Patient-Centered Care Framework. British Columbia Ministry of Health; 2015.

4. (29) Byrne A-L, Baldwin A, Harvey C. Whose centre is it anyway? Defining person-centred care in nursing: An integrative review. PLoS One. 2020;15:e0229923.

5. (40) Castro EM, Van Regenmortel T, Vanhaecht K, Sermeus W, Van Hecke A. Patient empowerment, patient participation and patient-centeredness in hospital care: A concept analysis based on a literature review. Patient Educ Couns. 2016;99:1923–39.

6. (42) Evans JM, Matheson G, Buchman S, MacKinnon M, Meertens E, Ross J, et al. Integrating cancer care beyond the hospital and across the cancer pathway: a patient-centred approach. Healthc Q. 2015;17 Spec No:28–32.

7. (17) Giusti A, Nkhoma K, Petrus R, Petersen I, Gwyther L, Farrant L, et al. The empirical evidence underpinning the concept and practice of person-centred care for serious illness: a systematic review. BMJ Glob Health. 2020;5:e003330.

8. (36) Greene SM, Tuzzio L, Cherkin D. A Framework for Making Patient-Centered Care Front and Center. Perm J. 2012;16:49–53.

9. (31) Holmström I, Röing M. The relation between patient-centeredness and patient empowerment: a discussion on concepts. Patient Educ Couns. 2010;79:167–72.

10. (19) Langberg EM, Dyhr L, Davidsen AS. Development of the concept of patient-centredness - A systematic review. Patient Educ Couns. 2019;102:1228–36.

11. Louw JM, Marcus TS, Hugo JFM. Patient- or person-centred practice in medicine? - A review of concepts. Afr J Prim Health Care Fam Med. 2017;9:e1–7.

12. (20) Mead N, Bower P. Patient-centredness: a conceptual framework and review of the empirical literature. Soc Sci Med. 2000;51:1087–110.

13. (10) Mitchell K-AR, Brassil KJ, Rodriguez SA, Tsai E, Fujimoto K, Krause KJ, et al. Operationalizing patient-centered cancer care: A systematic review and synthesis of the qualitative literature on cancer patients’ needs, values, and preferences. Psychooncology. 2020;29:1723–33.

14. (25) Oberst S, van Harten W, Saeter G, de Paoli P, Nagy P, Burrion J, Lovey J, Philip T. 100 European core quality standards for cancer care and research centres. 2020. 100 European core quality standards for cancer care and research centres [accessed 2025-05-29]

15. (32) Olson AW, Stratton TP, Isetts BJ, Vaidyanathan R, C Van Hooser J, Schommer JC. Seeing the Elephant: A Systematic Scoping Review and Comparison of Patient-Centeredness Conceptualizations from Three Seminal Perspectives. J Multidiscip Healthc. 2021;14:973–86.

16. (33) Ouwens M, Hermens R, Hulscher M, Vonk-Okhuijsen S, Tjan-Heijnen V, Termeer R, et al. Development of indicators for patient-centred cancer care. Support Care Cancer. 2010;18:121–30.

17. (39) Pel E, Engelberts I, Schermer M. Diversity of interpretations of the concept “patient-centered care for breast cancer patients”; a scoping review of current literature. J Eval Clin Pract. 2021. https://doi.org/10.1111/jep.13584.

18. (34) Robinson JH, Callister LC, Berry JA, Dearing KA. Patient-centered care and adherence: definitions and applications to improve outcomes. J Am Acad Nurse Pract. 2008;20:600–7.

19. (23) Santana MJ, Manalili K, Jolley RJ, Zelinsky S, Quan H, Lu M. How to practice person-centred care: A conceptual framework. Health Expect. 2018;21:429–40.

20. (35) Scholl I, Zill JM, Härter M, Dirmaier J. An integrative model of patient-centeredness - a systematic review and concept analysis. PLoS One. 2014;9:e107828.

21. (38) Symingtom D. Person Centred Health and Care Framework 2018-2021. NHS Eileanan Siar Western Isles; 2017.

22. (24) WHO. People-centred health care : a policy framework. 2007. https://www.who.int/publications-detail-redirect/9789290613176. Accessed 10 May 2023.

23. (37) Zucca A, Sanson-Fisher R, Waller A, Carey M. Patient-centred care: making cancer treatment centres accountable. Support Care Cancer. 2014;22:1989–97.
