# Supplementary material for: Practical Approaches to Patient-Centered Care in Europe: Mixed Methods Study Developing a Conceptual Framework for Comprehensive Cancer Care Networks
Source: JMIR Cancer. 2025 Jul 31;11:e59683. doi: 10.2196/59683 (PMC12355145; doi:10.2196/59683)
Supplement: Multimedia Appendix 3 [file cancer_v11i1e59683_app3.pdf]

Dear CraNE member,

as a central part of task 4 in WP6, we would like to reach a consensus on what patient-centeredness means in comprehensive cancer care networks* (CCCNs). For this purpose, we have developed a survey based on a systematic meta-review of generic and oncology-specific dimensions of patient-centeredness.

Based on this review, 8 dimensions were identified. Each dimension is composed of subgroups and concrete activities that can be implemented in care. In the following, we will present the proposed model to you in general and then take you through the 8 dimensions individually. For each dimension, we will ask you to rate its importance in CCCNs, brainstorm exemplary activities and share your impression on the current state-of-practice of patient-centered care in CCCNs. Finally, a working definition of patient-centered care in CCCNs is proposed. Filling out the survey may take approximately 20 to 30 minutes.

Thank you in advance for your time and participation!

Kind regards from Dresden,

Emily, Peggy, and Hannes

***A CCCN consists of multiple units belonging to different institutions dedicated to research, prevention, diagnosis, treatment, follow-up, supportive and palliative care and rehabilitation for the benefit of cancer patients and cancer survivors. Further information can be retrieved from the European Guide on Quality Improvement in Comprehensive Cancer Control.**

Section A: Dimensions of Patient-Centered Care in Comprehensive Cancer Care Networks

The following proposed model illustrates the 8 discerned dimensions of patient-centered care and their respective subgroups. Performance monitoring and support through digital health technologies were identified as overarching themes in all dimensions. Furthermore, the dimensions hold relevance in all stages of the patient pathway, even though certain dimensions may assume greater prominence in specific stages. Please take a moment to consider the proposed model.

Empowering Patients: Empowering patients refers to the process of enhancing patients' knowledge, skills, and self-awareness while also fostering their confidence to actively participate in care.

Engaging and Involving Patients: Engaging and involving patients refers to the active role of patients in healthcare, either independently through self-management activities or in collaboration with healthcare professionals through shared decision-making and care planning.

Treating the Patient as a Unique Person: Treating the patient as a unique individual entails acknowledging the patient's distinctive needs, preferences, values, feeling, beliefs, concerns, ideas, and expectations. An effort is made to get to know the patient, and care is personalized respectively.

Enhancing the Therapeutic Relationship: Enhancing the therapeutic relationship involves placing significant value on the relationship between caregivers and patients, with a focus on building a constructive partnership that prioritizes effective communication and is grounded in trust, respect, and compassion.

Providing Holistic Care: Providing holistic care emphasizes the importance of addressing not only patients' physical needs but also their emotional, social, and spiritual needs, recognizing the interconnections among these domains in shaping patients' health outcomes.

Enhancing a Patient-Centered Culture: Enhancing a patient-centered culture emphasizes the need for a profound cultural transformation, whereby patient-centeredness is deeply embedded in the underlying philosophy and organizational structures of the healthcare network.

Recognizing and Supporting the Caregiver as a Person: Recognizing and supporting the caregiver as a person emphasizes the significance of acknowledging the pivotal role of caregivers, their distinctive challenges, and providing them with multi-faceted support, including emotional, informational, and instrumental assistance.*

*Caregiver does not only relate to physicians and nurses but to all staff involved in the care of a patient, for example, including organizational support that may be offered by a case manager or navigation assistance.

Coordinating Care: Coordinating care highlights the need to optimize care processes, promote access, and ensure continuity of care for efficient and smooth care management that prioritizes patient needs and outcomes.

A1. Please indicate how important you feel the following dimensions are in CCCNs.

	Not important	Slightly important	Moderatly important	Import ant	Very important
Empowering Patients	☐	☐	☐	☐	☐
Engaging and Involving Patients	☐	☐	☐	☐	☐
Treating the Patient as a Unique Person	☐	☐	☐	☐	☐
Enhancing the Therapeutic Relationship	☐	☐	☐	☐	☐
Enhancing a Patient-Centered Culture	☐	☐	☐	☐	☐

	Not important	Slightly important	Moderatly important	Import ant	Very important
Providing Holistic Care	☐	☐	☐	☐	☐
Recognizing and Supporting the Caregiver as a Person	☐	☐	☐	☐	☐
Coordinating Care	☐	☐	☐	☐	☐

A2. What do you feel are the three most important dimensions of patient-centeredness in CCCNs?

Empowering Patients	☐
Engaging and Involving Patients	☐
Treating the Patient as a Unique Person	☐
Enhancing the Therapeutic Relationship	☐
Enhancing a Patient-Centered Culture	☐
Providing Holistic Care	☐
Recognizing and Supporting the Caregiver as a Person	☐
Coordinating Care	☐

A3. To what extent do you believe the following dimensions of patient-centered care are already implemented in CCCNs?

	To no extent	To little extent	To some extent	To a large extent	To a very large extent
Empowering Patients	☐	☐	☐	☐	☐
Engaging and Involving Patients	☐	☐	☐	☐	☐
Treating the Patient as a Unique Person	☐	☐	☐	☐	☐
Enhancing the Therapeutic Relationship	☐	☐	☐	☐	☐
Enhancing a Patient-Centered Culture	☐	☐	☐	☐	☐
Providing Holistic Care	☐	☐	☐	☐	☐
Recognizing and Supporting the Caregiver as a Person	☐	☐	☐	☐	☐
Coordinating Care	☐	☐	☐	☐	☐

Section B: Dimension: Empowering Patients

The dimension "Empowering Patients" is made up of the three subgroups: Information, Education and Motivation. Please consider the following graphic to gain a deeper insight into these subgroups.

B1. Please indicate how important you feel the following subgroups are in CCCNs.

	Not important	Slightly important	Moderatly important	Import ant	Very important
Information	☐	☐	☐	☐	☐
Education	☐	☐	☐	☐	☐
Motivation	☐	☐	☐	☐	☐

B2. What concrete activities, such as the examples given above, could be (or are being) performed in a CCCN-context to empower patients?

These activities could, for example, occur directly between caregiver and patient, in an individual CCCN unit or in relation to the organization of the network.

B3. To what extent do you believe the following subgroups of patient-centered care are already implemented in CCCNs?

	To no extent	To little extent	To some extent	To a large extent	To a very large extent
Information	☐	☐	☐	☐	☐
Education	☐	☐	☐	☐	☐
Motivation	☐	☐	☐	☐	☐

Section C: Dimension: Engaging and Involving Patients

The dimension "Engaging and Involving Patients" is made up of the three subgroups: Participation in care planning, Shared decision-making and Self-management. Please consider the following graphic to gain a deeper insight into these subgroups.

C1. Please indicate how important you feel the following subgroups are in CCCNs.

	Not important	Slightly important	Moderatly important	Import ant	Very important
Participation in care planning	☐	☐	☐	☐	☐

	Not important	Slightly important	Moderatly important	Import ant	Very important
Self-management	☐	☐	☐	☐	☐
Shared decision-making	☐	☐	☐	☐	☐

C2. What concrete activities, such as the examples given above, could be (or are being) performed in a CCCN-context to engage patients?

These activities could, for example, occur directly between caregiver and patient, in an individual CCCN unit or in relation to the organization of the network.

C3. To what extent do you believe the following subgroups of patient-centered care are already implemented in CCCNs?

	To no extent	To little extent	To some extent	To a large extent	To a very large extent
Participation in care planning	☐	☐	☐	☐	☐
Self-management	☐	☐	☐	☐	☐
Shared decision-making	☐	☐	☐	☐	☐

Section D: Dimension: Treating the Patient as a Unique Person

The dimension "Treating the Patient as a Unique Person" is made up of the two subgroups: Knowing the patient and Personalization of healthcare. Please consider the following graphic to gain a deeper insight into these subgroups.

D1. Please indicate how important you feel the following subgroups are in CCCNs.

	Not important	Slightly important	Moderatly important	Import ant	Very important
Knowing the patient	☐	☐	☐	☐	☐
Personalization of healthcare	☐	☐	☐	☐	☐

D2. What concrete activities, such as the examples given above, could be (or are being) performed in a CCCN-context to ensure that the patient is treated as a unique person?

These activities could, for example, occur directly between caregiver and patient, in an individual CCCN unit or in relation to the organization of the network.

D3. To what extent do you believe the following subgroups of patient-centered care are already implemented in CCCNs?

	To no extent	To little extent	To some extent	To a large extent	To a very large extent
Knowing the patient	☐	☐	☐	☐	☐
Personalization of healthcare	☐	☐	☐	☐	☐

Section E: Dimension: Enhancing the Therapeutic Relationship

The dimension "Enhancing the Therapeutic Relationship" is made up of the three subgroups: Communication, Respect and compassion and Trust. Please consider the following graphic to gain a deeper insight into these subgroups.

E1. Please indicate how important you feel the following subgroups are in CCCNs.

	Not important	Slightly important	Moderatly important	Import ant	Very important
Communication	☐	☐	☐	☐	☐
Respect and compassion	☐	☐	☐	☐	☐
Trust	☐	☐	☐	☐	☐

E2. What concrete activities, such as the examples given above, could be (or are being) performed in a CCCN-context to enhance the therapeutic relationship?

These activities could, for example, occur directly between caregiver and patient, in an individual CCCN unit or in relation to the organization of the network.

E3. To what extent do you believe the following subgroups of patient-centered care are already implemented in CCCNs?

	To no extent	To little extent	To some extent	To a large extent	To a very large extent
Communication	☐	☐	☐	☐	☐
Respect and compassion	☐	☐	☐	☐	☐
Trust	☐	☐	☐	☐	☐

Section F: Dimension: Enhancing a Patient-Centered Culture

The dimension "Enhancing a Patient-Centered Culture" is made up of the three subgroups: Philosophy, Organization and Teamwork. Please consider the following graphic to gain a deeper insight into these subgroups.

F1. Please indicate how important you feel the following subgroups are in CCCNs.

	Not important	Slightly important	Moderatly important	Import ant	Very important
Philosophy	☐	☐	☐	☐	☐
Organization	☐	☐	☐	☐	☐
Teamwork	☐	☐	☐	☐	☐

F2. What concrete activities, such as the examples given above, could be (or are being) performed in a CCCN-context to enhance a patient-centered culture?

These activities could, for example, occur directly between caregiver and patient, in an individual CCCN unit or in relation to the organization of the network.

F3. To what extent do you believe the following subgroups of patient-centered care are already implemented in CCCNs?

	To no extent	To little extent	To some extent	To a large extent	To a very large extent
Philosophy	☐	☐	☐	☐	☐
Organization	☐	☐	☐	☐	☐
Teamwork	☐	☐	☐	☐	☐

Section G: Dimension: Providing Holistic Care

The dimension "Providing Holistic Care" is made up of the three subgroups: Integrating medical and non-medical care, Emotional and social support and Family and friends. Please consider the following graphic to gain a deeper insight into these subgroups.

G1. Please indicate how important you feel the following subgroups are in CCCNs.

	Not important	Slightly important	Moderatly important	Import ant	Very important
Integrating medical and non-medical care	☐	☐	☐	☐	☐
Emotional and social support	☐	☐	☐	☐	☐
Family and friends	☐	☐	☐	☐	☐

G2. What concrete activities, such as the examples given above, could be (or are being) performed in a CCCN-context to provide holistic care?

These activities could, for example, occur directly between caregiver and patient, in an individual CCCN unit or in relation to the organization of the network.

G3. To what extent do you believe the following subgroups of patient-centered care are already implemented in CCCNs?

	To no extent	To little extent	To some extent	To a large extent	To a very large extent
Integrating medical and non-medical care	☐	☐	☐	☐	☐
Emotional and social support	☐	☐	☐	☐	☐
Family and friends	☐	☐	☐	☐	☐

Section H: Dimension: Recognizing and Supporting the Caregiver as a Person

The dimension "Recognizing and Supporting the Caregiver as a Person" is made up of the three subgroups: Personal qualities, Training and qualification and Support. Please consider the following graphic to gain a deeper insight into these subgroups.

H1. Please indicate how important you feel the following subgroups are in CCCNs.

	Not important	Slightly important	Moderatly important	Import ant	Very important
Personal qualities	☐	☐	☐	☐	☐
Training and qualification	☐	☐	☐	☐	☐
Support	☐	☐	☐	☐	☐

H2. What concrete activities, such as the examples given above, could be (or are being) performed in a CCCN-context to ensure that the caregiver is recognized and supported as a person?

These activities could, for example, occur directly between caregiver and patient, in an individual CCCN unit or in relation to the organization of the network.

H3. To what extent do you believe the following subgroups of patient-centered care are already implemented in CCCNs?

	To no extent	To little extent	To some extent	To a large extent	To a very large extent
Personal qualities	☐	☐	☐	☐	☐
Training and qualification	☐	☐	☐	☐	☐
Support	☐	☐	☐	☐	☐

Section I: Dimension: Coordinating Care

The dimension "Coordinating Care" is made up of the three subgroups: Access to care, Continuity of care and Optimization of processes. Please consider the following graphic to gain a deeper insight into these subgroups.

I1. Please indicate how important you feel the following subgroups are in CCCNs.

	Not important	Slightly important	Moderatly important	Import ant	Very important
Access to care	☐	☐	☐	☐	☐
Continuity of care	☐	☐	☐	☐	☐
Optimization of processes	☐	☐	☐	☐	☐

I2. What concrete activities, such as the examples given above, could be (or are being) performed in a CCCN-context to coordinate care?

These activities could, for example, occur directly between caregiver and patient, in an individual CCCN unit or in relation to the organization of the network.

I3. To what extent do you believe the following subgroups of patient-centered care are already implemented in CCCNs?

	To no extent	To little extent	To some extent	To a large extent	To a very large extent
Access to care	☐	☐	☐	☐	☐
Continuity of care	☐	☐	☐	☐	☐
Optimization of processes	☐	☐	☐	☐	☐

Section J: Definition of patient-centeredness in a comprehensive cancer care network (CCCN)

J1. **In case you thought of any further examples for patient-centered activities in a CCCN setting, which were maybe difficult to sort into one of the described dimensions, please use the space provided to let us know about them.**

J2. **Based on the meta-review, we propose the following definition for patient-centeredness in comprehensive cancer care networks (CCCNs):**

Patient-centeredness in CCCNs is a philosophy of care prioritizing cancer patient's physical, emotional, and social needs, as well as personal values on every step of the patient pathway. In patient-centered cancer care networks patients are empowered and engaged to become active partners in healthcare in relation to their individual preferences with the goal of providing personalized, high-quality, holistic care with the best possible outcomes.

	Strongly disagree	Disagree	Neither agree nor disagree	Agree	Strongly agree
To what extent do you agree or disagree with the proposed definition?	☐	☐	☐	☐	☐

J3. **Please explain your reasoning concerning the extent that you agree or disagree with the proposed defintion of patient-centeredness in CCCNs.**

In case an important aspect of patient-centeredness in CCCNs is missing to you in the proposed definitiion, please let us know which.

J4. **Do you agree to the proposed model of patient-centeredness in CCCNs?**

Yes ☐

No ☐

J5. **Please explain why you do not agree with the proposed model of patient-centeredness in CCCNs.**

In case an important aspect of patient-centeredness in CCCNs is missing to you in the proposed model, please let us know which.

J6. **Please indicate the stakeholder group or organization to which you would assign yourself.**

- Health care provision ☐
- Research ☐
- Network management and certification ☐
- Cancer organization ☐
- Ministry of health ☐
- Cancer patient organization ☐
- Public health institute ☐
- Other ☐

Other

Thank you very much for participating! We will be happy to share the results with you soon!

Kind regards,

Emily, Peggy and Hannes

Sources used in review process:

1. Santana MJ, Manalili K, Jolley RJ, Zelinsky S, Quan H, Lu M. How to practice person-centred care: A conceptual framework. Health Expect. 2018;21:429–40.

2. Byrne A-L, Baldwin A, Harvey C. Whose centre is it anyway? Defining person-centred care in nursing: An integrative review. PLoS One. 2020;15:e0229923.

3. The British Columbia Patient-Centered Care Framework. British Columbia Ministry of Health; 2015.

4. Scholl I, Zill JM, Härter M, Dirmaier J. An integrative model of patient-centeredness - a systematic review and concept analysis. PLoS One. 2014;9:e107828.

5. Olson AW, Stratton TP, Isetts BJ, Vaidyanathan R, C Van Hooser J, Schommer JC. Seeing the Elephant: A Systematic Scoping Review and Comparison of Patient-Centeredness Conceptualizations from Three Seminal Perspectives. J Multidiscip Healthc. 2021;14:973–86.

6. American Geriatrics Society Expert Panel on Person-Centered Care. Person-Centered Care: A Definition and Essential Elements. J Am Geriatr Soc. 2016;64:15–8.

7. Robinson JH, Callister LC, Berry JA, Dearing KA. Patient-centered care and adherence: definitions and applications to improve outcomes. J Am Acad Nurse Pract. 2008;20:600–7.

8. Brickley B, Sladdin I, Williams LT, Morgan M, Ross A, Trigger K, et al. A new model of patient-centred care for general practitioners: results of an integrative review. Fam Pract. 2020;37:154–72.

9. Holmström I, Röing M. The relation between patient-centeredness and patient empowerment: a discussion on concepts. Patient Educ Couns. 2010;79:167–72.

10. People-centred health care: a policy framework. World Health Organization; 2013.
