# Supplementary material for: Practical Approaches to Patient-Centered Care in Europe: Mixed Methods Study Developing a Conceptual Framework for Comprehensive Cancer Care Networks
Source: JMIR Cancer. 2025 Jul 31;11:e59683. doi: 10.2196/59683 (PMC12355145; doi:10.2196/59683)
Supplement: Multimedia Appendix 4 [file cancer_v11i1e59683_app4.docx]

# Appendix 4: Dimensions and possible activities of patient-centred care in CCCNs

The table provided below presents the dimensions and subdimensions that align with the proposed framework of patient-centeredness in CCCNs, along with their descriptions. Furthermore, concrete activities that were gathered during the review and survey process, which have the potential to facilitate the practical implementation of each subcategory, are listed under their respective subcategories. Please note that the cells highlighted in light red indicate that at least one of the sources within that activity pertains to an oncology setting. Writing in blue color was added during the focus groups performed with patient representatives. Furthermore, it is important to note that certain activities may be relevant to multiple dimensions, and there are clear synergies and overlaps between the different categories. Therefore, a primary allocation has been made to assign activities to specific subdimensions based on their primary focus.

| **Nr.** | **Dimension** | **Description/ Tool Support** | **Sources** |
| --- | --- | --- | --- |
| **1** | **Empowering patients**  Empowering patients refers to the process of enhancing patients' knowledge, skills, and self-awareness while also fostering their confidence to actively participate in care. | | [1–20] |
| **1.1** | **Subtheme: Information**  *Patients are actively provided with and have access to tailored information.* | | [1–13, 17, 20] |
|  | Share (tailored) information with the patient | - Timely, accurate, and complete information sharing - Availability in different languages - For example, about condition, own influence on condition, care stages and process, individual and diagnosis-specific characteristics of illness, what to expect during and after treatment, minimizing costs, nutrition, prognosis, side-effects/ symptoms, treatment options and rationale, updates on condition and treatment etc. | [1–12, 21] |
|  | Provide procedures to specify how and by whom patients are informed | - Ensure that patients have a fixed contact person | [21] |
|  | Ensure access to care plans/ health records for patients | - All programs/software should potentially also be usable by patients, check for compatibility with different hardware/ systems - Also documents from the tumor board | [1, 10] |
|  | Give access to audio recordings and written summaries of clinical consultation if appropriate |  | [10] |
|  | Provide written information in conjunction with verbal information during consultations | - During consultation provide patients with the information about a contact person, who they can contact, if questions arise later on | [10]  Survey |
|  | Provide sufficient time with healthcare providers to ensure that there was enough time to provide all information, answer all questions, explore worries and concerns |  | Survey |
|  | Inform patients about possibilities to be involved in clinical trials | - These possibilities should be realistic for patients and further (understandable) information should be retrievable from the website of the house or from a different information source - Desirable would be a solution similar to clinicaltrials.gov, but presented in a way that is understandable for patients and available in different languages. | Survey |
|  | Provide a website with information on the CCCN | - Navigation support - Information on healthcare providers - Units, Patient advisory council etc. | Survey |
|  | Make an information and support center available and easily accessible for staff, patients, family members and caregivers | - Inform about the information and support center | [21] |
|  | Make sure patients understand the provided information |  | Survey |
| **1.2** | **Subtheme: Education**  *Patients are educated about their disease, including skill-building activities.* | |  |
|  | Provide health education/ management programs  Perform online evidence-based health education | - Possibly also on soft skills, such as communication for patients | [1–12] Survey  [10, 21] |
|  | Make use of waiting rooms and other public spaces for opportunistic health education | - A designated individual should be responsible for a well-targeted collection of brochures and up-to-date informational materials - Actively curate a diverse selection of providers and advocate for the visibility of rare disease - Exercise caution in accessing materials when the quantity is limited, and ensure strategic distribution to maximize impact | [10] |
|  | Ensure access to patient advocacy/ advisory services |  | [1, 11] |
|  | Make patient discussion groups available, joint offers with self-help | - These should be as disease specific as possible | Survey |
| **1.3** | **Subtheme: Motivation**  *Patients are motivated to become empowered and engaged in their healthcare.* | |  |
|  | Motivate patients to inform themselves and be involved/ engaged in their care | - Provide contact points, patient advisory board - In relation to capabilities | [9] |

| **Nr.** | **Dimension** | **Description** | **Sources** |
| --- | --- | --- | --- |
| **2** | **Engaging and Involving Patients**  Engaging and involving patients refers to the active role of patients in healthcare, either independently through self-management activities or in collaboration with healthcare professionals through shared decision-making and care planning. | | [1–20] |
| **2.1** | **Subtheme: Self-Management**  *Recognition and active support of the patient’s ability and responsibility to self-manage his or her disease.* | | [1, 2, 4, 5, 8, 10, 11, 13–15] |
|  | Provide patients with self-management tools | - For example, patient passports for recording their cancer journey - Include regular evaluations with the patients to track progress and enhance motivation | [1, 2, 8, 10, 14, 15]  Survey |
|  | Provide self-management training programs for patients | - Online or in person | [1, 2, 8, 10, 14, 15] |
|  | Provide patients with navigation assistance | - In the healthcare system - Along their patient journey - Provide patients with information on contact options to the navigation assistance | [11, 14] |
|  | Design healthcare facilities and services providing navigation |  | [1, 14] |
|  | Support self-management of pain |  | Survey |
|  | Initiate specific engagement programs for patients | Ex. Self-initiated follow up for patients, life after cancer – enhancing survivorship, cancer thriving and surviving | Survey |
| **2.2** | **Subtheme: Participation in care planning**  *Active participation of and collaboration with patients under consideration of patient's preferences for involvement.* | | [1–5, 8, 9, 13, 16] |
|  | Patients are given the opportunity to: i) say what and who matters to them ii) are supported to ensure this is achieved iii) have this regularly reviewed | - Create opportunities for integrating the needs and expectations of patients and families - Provide patient-friendly information/ communication to support choices, show available options | [2, 4] |
|  | Co-designing and/ or discussing care plans with patients | - Finding common ground on what the problem is and agreeing on a management | [1, 9]  Survey |
|  | Set joint goals with patients |  | [1] |
|  | Stimulate patients to ask questions | - Provide patients with possible questions that may be relevant (Tool: Question Prompt Sheets) | [8]  Survey |
|  | Find out and respect the degree to which patients want to be and can be involved in care planning |  | [15] |
| **2.3** | **Subtheme: Shared decision-making**  *Patients and caregivers work together to reach the best possible decision for the patient.* | | [1, 2, 5–8, 10, 14–17] |
|  | Physicians and medical staff recognize the patient as competent to make decisions and value their lived experience |  | [2] |
|  | Perform shared/ informed decision-making | - Ensures SDM on basis of best-available evidence - Couple with patient preferences for SDM | [1, 5–8, 14–17] Survey |
|  | Offer a clinical decision support/ shared decision-making systems | - No "persuasion" to clinical decisions or for research, instead better information on necessities | [5, 14] |
|  | Provide communication and negotiation skills trainings to patients that lead to meaningful participation in decision-making |  | [10] Survey |
|  | Provide decision-making tools |  | [7, 10, 16, 17] |
|  | Exchange clinical and experimental knowledge | - Between doctors, nursing staff, other patients, and the patient | [13] |
|  | Find out and respect the degree to which patients want to be involved in decision-making processes | - 40% to 64% mismatch between preference and actual experience of decision making - Provide detailed information beforehand | [15] |
| **2.4** | **Subtheme: Co-creation of services and systems**  *Patients should be provided with opportunities to support the design and make improvements to healthcare services and systems.* | | Survey |
|  | Co-create patient information with patients and/or patient representatives |  | Survey |
|  | Co-create care pathways |  | Survey |
|  | Give patients the possibility to speak out |  | Survey |
|  | Involve patients, their organizations and support groups in the planning and organization of services | - Develop healthcare professional and patient groups who work together as one group to develop services based on patient needs - For example, specific survivorship groups - Encourage patients to network on an EU and global level to identify approaches in other centers that may be suitable. | [21]  Survey |
|  | Organize public and patient involvement events, panels, and committees |  | Survey |
|  | Engage patients in strategic planning, design of guidelines and policy |  | [3]  Survey |
|  | Develop (disease specific) patient representative groups within a healthcare institution/ network | - Can address clinical, operational, and research activities - Integrate caregivers in representative groups - Organize a separate group for relatives - Ensure that they are represented in relevant activities | Survey |
|  | Advertise and promote work and support given by patient representatives | - Ensure that staff know about and are confident to reach out, ask for patient support in projects - Actively utilize support by patients in projects | Survey |
|  | Offer participatory events, meetings, publications about current programs or projects inside the CCCN | - Development of networking days, where patients and caregivers/ staff attend and participate together | Survey |
|  | Embed patients at key stages in the hierarchical governance structure of the CCCN | - The level of formal obligation that patients have/ should receive in supporting governance structures should be addressed | Survey |
|  | Provide an infrastructure with an explicit representative to support the development of volunteering | - Support networking of full-time staff with volunteers - Minimize the burden on volunteers | [10, 16]  Survey |

| **Nr.** | **Dimension** | **Description** | **Sources** |
| --- | --- | --- | --- |
| **3** | **Enhancing the therapeutic alliance**  Enhancing the therapeutic relationship involves placing significant value on the relationship between caregivers and patients, with a focus on building a constructive partnership that prioritizes effective communication and is grounded in trust, respect, and compassion. | | [1–8, 10–14, 16, 18–20, 22] |
| **3.1** | **Subtheme: Clinician-patient communication**  *Communication and understanding between patients and caregivers is fostered.* | | [1–8, 10–14] |
|  | Listen carefully / Active listening |  | [1–3, 6, 8, 13, 14] Survey |
|  | Answer questions |  | [11] |
|  | Communicate clearly in relation to patient’s needs and capabilities | - Cancellations should be clearly communicated and explained | [14] |
|  | Communicate timely |  | [11] |
|  | Talk with (not to or about) the patient |  | [11] |
|  | Use non-verbal communication |  | [8] |
|  | Take your time |  | [5, 8]  Survey |
|  | Provide concise, simple, harmonized, and effective communication and counselling protocols for health practitioners |  | [10]  Survey |
| **3.2** | **Subtheme: Respectful and compassionate care**  *Patients are treated with respect and compassion.* | | [1, 3, 7, 8, 11–14, 16] |
|  | Be empathetic |  | [14] |
|  | Be warm, friendly, and approachable |  | [11, 16] Survey |
|  | Be interested in the patient |  | [8] |
|  | Introduce yourself |  | [16] |
|  | Be polite |  | [16] |
|  | Treat patients with dignity and respect |  | [7, 11, 13, 16] |
| **3.3** | **Subtheme: Trusting relationship**  *Trustful patient-caregiver relationships are sought and strengthened.* | | [1, 2, 6, 8, 11, 13, 14, 16, 22] |
|  | Be sincere and truthful |  | [2, 8, 11, 13, 14] |
|  | Ensure one primary or lead point of contact for the patient on the healthcare team |  | [6, 8] Survey |
|  | Structure appointment system to allow patients to see the same professional over time |  | [22]  Survey |
|  | Be professional |  | [16] |
|  | Check in on patients |  | [11] |
|  | Protect patients’ privacy | - In consultation/ examinations - When communicating | [8, 11, 16] |
|  | Design spaces and health care facilities, so that privacy is protected |  | [1, 14] |
|  | Provide means for direct interaction between patients and healthcare providers |  | Survey |

| **Nr.** | **Dimension** | **Description** | **Sources** |
| --- | --- | --- | --- |
| **4** | **Treating the Patient as a unique person**  Treating the patient as a unique individual entails acknowledging the patient's distinctive needs, preferences, values, feeling, beliefs, concerns, ideas, and expectations. An effort is made to get to know the patient, and care is personalized respectively. | | [1, 2, 4–9, 11, 13–15, 17–20, 22, 23] |
| **4.1** | **Subtheme: Knowing the patient**  *Caregivers strive to get to know their patients in relation to their individual circumstances.* | | [6–8, 11, 14, 17] |
|  | Create a dialogue with patients to develop understanding of their situation in the context of their everyday lives |  | [8]  Survey |
|  | Perform a holistic needs assessment to elicit patients’ priorities |  | Survey |
|  | Continuously review patients’ goals in relation to their current care plan |  | [6] |
|  | Also address patient needs, not only the disease |  | [17] |
|  | Find common ground based on patient preferences |  | [7, 14] |
|  | Respect the patient as a person with unique preferences, values, and needs |  | [11] |
|  | Provide time before consultations for physicians to prepare |  | Survey |
| **4.2** | **Subtheme: Personalization of healthcare**  *Healthcare provision is personalized to patients’ circumstances and preferences* and based on the best-available evidence. | | [1, 2, 5, 6, 8, 9, 11, 14, 15, 17, 20, 22, 23] Survey |
|  | Create processes and structures that allow flexibility in service delivery and care practice |  | [22] |
|  | Create an individualized care plan based on patients’ goals and preferences | - Also consider mobility, hospital-home distance, family and support | [6]  Survey |
|  | Use knowledge of the patient as a unique person for effective interactions  Be responsive to patients’ individual preferences, values, and needs | - If possible, also include brief conversation about the patient's private life | [8, 14]  [1, 5, 8, 9, 11, 17] |
|  | Create partnerships with community organizations | - Patients may (if possible) prefer the convenience of home- or community-based care | [1, 23] |
|  | If possible, enable home care possibilities | - Patients may (if possible) prefer the convenience of home- or community-based care | [23] |

| **Nr.** | **Dimension** | **Description** | **Sources** |
| --- | --- | --- | --- |
| **5** | **Providing Holistic Care**  *Providing holistic care emphasizes the vital role of addressing not only the physical requirements of patients but also their emotional, social, and spiritual needs.* | | [1–5, 10–16, 18–20, 22, 23] |
| **5.1** | **Subtheme: Integrating medical, specialized and supportive care**  *Medical, specialized and supportive care are recognized as interconnected dimensions in shaping patients’ overall health outcomes.* | | [1, 4, 5, 11–13, 20, 22, 23] |
|  | Ensure an early identification of patients who can benefit from palliative care | - Complex health and social needs at the end of life, if not adequately addressed, result in poor patient outcomes and experiences, as well as high costs to the system due to inappropriate utilization of acute care services - Despite its significant potential cancer patients continue to be referred to palliative care programs in low numbers and late in their cancer journey - Network of Social Services, Palliative Care, Hospice | [15, 23] |
|  | Address survivorship needs | - 30 to 50% of posttreatment cancer survivors report unmeet information needs - Provide tailored and culturally sensitive survivorship care - Address fear of cancer reoccurrence - Understand what is important from the standpoint of the survivor (ex. “right to be forgotten”) | [15, 17, 23] |
|  | Provide specialized care | - Specialized care plays a crucial role in tailoring interventions and treatments to specific medical conditions, ensuring precision and effectiveness in addressing the unique healthcare needs of individual patients | Survey |
|  | Provide access to supportive care services | - For example, rehabilitation, ambulatory services, hearing aid manufacturer, social services, physiotherapy, acupuncture, hypnosis, meditation etc. - Different access options in different public health systems | Survey |
|  | Promote continuation of normality and self-identity | - Participating in regular personal life activities - Providing meaningful activities for inpatients | [22] |
|  | Ensure best possible physical comfort | - Pain, fatigue - Holistic pain management | [15] |
|  | Support patients’ management of symptoms, side-effects, and co-morbidities | - If possible and wanted, enable care at home | Survey |
|  | Provide relevant support | - Could, for example, be from a social worker or a patient association/ charity - Also, contacts to service providers (wig makers etc.) | Survey |
| **5.2** | **Subtheme: Emotional and social support**  *Emotional and social support is repeatedly offered to patients.* | | [1, 4, 5, 10, 11, 13–16] |
|  | Provide a supportive and accommodating environment | - Environments that are welcoming, comfortable, and respectful - An organizational culture that respects everyone’s values and choices | [1, 10, 13, 14] |
|  | Provide contacts and funding for self-help programs |  | [10] |
|  | Offer religious spaces |  | [1, 14] |
|  | Refer to appropriate (cancer-specific, if necessary) patient or peer support/ advisory groups |  | [10, 11] Survey |
|  | Use social prescribing | - An approach that connects people to activities, groups, and services in their community to meet the practical, social and emotional needs that affect their health and wellbeing | Survey |
|  | Ensure that patients are offered spiritual care support |  | [16] |
|  | Provide emotional support | - Distress, anxiety, depression | [4, 5, 15] |
|  | Be sympathetically present |  | [4, 5, 15] |
|  | Offer access to onco-psychology services |  | Survey |
| **5.3** | **Subtheme: Family and Friends**  *Family/ friends are recognized and respected as central to patients' well-being.* | | [4, 5, 10–12, 14–16, 22] |
|  | Ensure that families are offered spiritual care support |  | [16] |
|  | Discuss which family members or/ and friends the patients want to be involved in their care and be responsive to wishes |  | [4, 5, 11, 12, 14, 16, 22]  Survey |
|  | Address the needs of family and friends | - For example, information needs | [22] |
|  | Offer appropriate and flexible visiting policies | - Possibly also for overnight stays | [10]  Survey |
|  | Facilitate healing relationships |  | [14] |
|  | Welcome participation of family and friends in consultations | - Arrange appointments accordingly | [15] |

| **Nr.** | **Dimension** | **Description** | **Sources** |
| --- | --- | --- | --- |
| **6** | **Recognizing and Supporting the Caregiver**^[[1]](#footnote-1)^ **as a Person**  *Recognizing and supporting the caregiver as a person emphasizes the significance of acknowledging the pivotal role of caregivers, their distinctive challenges, and providing them with multi-faceted support, including emotional, informational, and instrumental assistance.* | | [1, 2, 4, 5, 7, 8, 10, 14, 16, 18, 18, 19, 22] |
| **6.1** | **Subtheme: Personal qualities**  *The caregiver possesses personal qualities necessary to perform patient-centered care* | | [2, 4, 5, 8, 10, 18] |
|  | Commitment to job |  | [5] |
|  | Commitment to quality |  | [10] |
|  | Respect, Non-judgmental behavior |  | [2] |
|  | Self-reflective behavior | - Self-aware of own limitations - Clarity of own beliefs and values | [4, 5, 8] |
|  | Empathy, Compassion |  | [4, 8] |
|  | Honesty |  | [4] |
| **6.2** | **Subtheme: Training and qualification**  *Caregivers are continuously trained and qualified* | | [1, 2, 5, 7, 10, 14, 16, 22] |
|  | Ensure an appropriate skill mix |  | [5] |
|  | Encourage life-long learning, personal growth, and development  Provide continuing professional development possibilities in different formats | - Training also through internal exchange in the team, thus appreciation of special skill - External training offers the opportunity to exchange experiences across institutions, so that experiences can also be transferred across regions | [10]  [2, 5, 7, 10, 16] |
|  | Implementation of educational programs, standardized training |  | [1, 16] |
|  | Trainings in:   - Building a trustful relationship with patients - Leadership - Developing tailored interventions - Positive first impression - Confidentiality - Holistic perception of human organism - Patient-centeredness (interpersonal skills) - Communication (e.g., announcement of diagnosis) - Customer service training - Adaptation to different cultures - Self-care | - For all new physicians and retraining as needed based on patient rating of clinician communication. - Translation of education programs into practice through continued professional education and mentorship | [10, 14, 16, 22]  Survey |
|  | Implement professional education and accrediting bodies |  | [1, 16] |
|  | Promote evidence-based practice |  | [10] |
| **6.3** | **Subtheme: Support**  *Caregivers are secured and supported to enable them to perform their everyday tasks.* | | [1, 10, 16] |
|  | Instill importance of taking care of one's own health and well-being |  | [10] |
|  | Ensure continuous support to caregivers along the whole process of care |  | Survey |
|  | Ensure staffing standards | - Quantity and workforce mix for different levels and types of health facilities | [10]  Survey |
|  | Provide financial security |  | [10]  Survey |
|  | Ensure that staff are offered spiritual/ psychological care support |  | [16]  Survey |
|  | Provide performance-based incentive packages/ awards | - Possible, nominated by patients - Public acknowledgements of model staff - Ex. Daisy awards for nursing staff | [1, 10] Survey |
|  | Ensure that caregivers also have information detailing support systems available to them |  | Survey |
|  | Provide employee assistance programs |  | Survey |
|  | Provide leisure activities/ (discounted) sport programs for staff | - For example, staff choir - Consider networking with other institutions | Survey |
|  | Support transportation: car parking facilities, discounted fees for public transport, accommodation for staff traveling long distances |  | Survey |
|  | Support harmonization of work and family | - For example, childcare services | Survey |

| **Nr.** | **Dimension** | **Description** | **Sources** |
| --- | --- | --- | --- |
| **7** | **Coordinating Care**  *Coordinating care highlights the need to optimize care processes, promote access, and ensure continuity of care for an efficient and smooth care management that prioritizes patient needs and outcomes.* | | [1, 4–7, 10–15, 17, 18, 20, 22, 23] |
| **7.1** | **Subtheme: Access to care**  *Patients have timely and affordable access to care* | | [1, 4, 5, 10–12, 14, 15] |
|  | Secure e-mail access for patients to their clinicians |  | [14] |
|  | Enable direct access to specialty care clinicians |  | [14] |
|  | Ease appointment-making process  Suitable appointment scheduling | - Avoid longer waiting times | [10, 14, 15] |
|  | Affordability of care | - Including complimentary care and therapies, dental, pharma care, ambulance, financing nursing home | [1, 14] |
|  | Ensure easy access/ direct communication channels between patients and caregivers AND/ OR  Provide a distinct communication possibility to the service, which can forward queries from patients, so that they can be answered quickly | - Contact to clinical staff in case of emergencies | [21]  Survey |
|  | Ensure universal access to primary and secondary care |  | Survey |
| **7.2** | **Subtheme: Continuity of care**  *Coherent and seamless, high-quality delivery of care* | | [1, 4–6, 10–14, 22, 23] |
|  | Ensure interprofessional leadership teams |  | [10] |
|  | Implement patient pathways across different providers, establish cooperation pathways across specialisms and network partners | - Evaluate and update regularly to ensure access, continuity of care and optimization of processes - Seamless, loss-free, standardized communication pathways between all supply partners - Manage care transitions | [14, 22, 23] Survey |
|  | Organize services in networks/ hub and spoke model of care |  | Survey |
|  | Perform follow-ups after treatments and procedures |  | [11, 12] |
|  | Coordinate with community resources |  | [6, 14] |
|  | Ensure seamless flow of information between all relevant stakeholders |  | [1, 14, 23] |
|  | Establish communication protocols across disciplines |  | [10] |
|  | Introduce and strengthen models of care | - New models of care across services and settings | [10] |
|  | Provide a defined discharge procedure, including giving information of further treatment, follow-up, re-admission, and home care to patients  Provide discharge summaries and care plans |  | [21]  Survey |
|  | Involve and support the main family caregiver |  | Survey |
|  | Establish a nurse navigator/ contact nurse to coordinate the whole pathway of care |  | Survey |
|  | Perform a multidisciplinary tumor board to mutually decide on the treatment and next steps for patients | - Possibly with patients | Survey |
|  | Define wait times until diagnosis |  | Survey |
| **7.3** | **Subtheme: Optimization of processes**  *Processes are redesigned and optimized to enhance effectivity* | | [7, 10–12, 14, 17, 22] |
|  | Create protocols for discharge and referral |  | [10] |
|  | Work efficiently |  | [7, 14] |
|  | Pre-visit outreach to patients by medical assistance to ensure focus on important problems in encounter and that patients brings all relevant documents | - Provide pre-visit education/ information | [14]  Survey |
|  | Minimize clinical wait times |  | [14] |
|  | Dedicate case managers for cancer patients/ Oncology nurse with case management task |  | [12]  Survey |
|  | Send reminder notices for specific interventions |  | [10] |
|  | Ensure transparent, accessible, and understandable service protocols that improve patient flow |  | [10] |
|  | Lean process review all services in regular intervals with the aim of optimizing efficiency |  | Survey |
|  | Continuous quality improvement |  | [6] |

| **Nr.** | **Dimension** | **Description** | **Sources** |
| --- | --- | --- | --- |
| **8** | **Enhancing a Patient-Centered Culture**  *Enhancing a patient-centered culture emphasizes the need for a profound cultural transformation, whereby patient-centeredness is deeply embedded in the underlying philosophy and organizational structure of the healthcare network.* | | [1–6, 10, 12, 14, 16, 22] |
| **8.1** | **Subtheme: Philosophy**  *A philosophy of patient-centered care, reflected throughout the entire network* | |  |
|  | Position patient-centered care as a major priority in the CCCN |  | [2]  Survey |
|  | Open spaces for patient-centered care despite competing priorities |  | [2] |
|  | Ensure responsiveness of the entire care team to patient and family needs |  | [14] |
|  | Inculcate a culture of reflective practice |  | [10] |
|  | Corporate responsibility for equality and diversity of patients and staff |  | [16] |
|  | Establish trust and respect as core values of the CCCN |  | Survey |
|  | Establish a patient charter: key rights and responsibilities of people receiving care | - Emphasize patient-centered care as a patient’s right - Make certain that there is an accountable individual responsible for keeping the patient charter up-to-date and “alive” | Survey |
| **8.2** | **Subtheme: Organization**  *The network is organized in a manner supporting patient-centered care* | | [1–3, 5, 10, 16] |
|  | Establish services promoting a patient-centered culture | - Interpretation and language services, access to information | [1, 3, 5, 16] |
|  | Address systematic barriers to a patient-centered culture | - Leadership, policy, culture | [2, 5] |
|  | Implement organizational system support |  | [3, 5] |
|  | Support innovation and risk-taking potential |  | [5] |
|  | Introduce a strategic framework for patient-centeredness |  | [5] |
|  | Implement change management aimed an anchoring patient centered care in everyday culture | - Regular evaluation of the changes implemented. | [3] |
| **8.3** | **Subtheme: Teamwork**  *Teamwork, -building and –development are fostered throughout the network* | | [1, 3–6, 10, 12, 14, 16, 22] |
|  | Establish and strengthen a multidisciplinary and interprofessional patient care team | - Provide a detailed job description for each team member, incorporating duties and responsibilities as a team member - Make sure that teams are flexible in composition and adaptable to changes in patients’ health statuses, circumstances, and goals | [6, 10, 12] |
|  | Encourage teamwork and team building | - Organize team events - Share power in the team, also between the hierarchies - Share responsibilities | [1, 5] |
|  | Make patients into integral team members |  | [6] |
|  | Ensure strong team leadership committed to a patient-centered culture |  | [3, 16, 22] |
|  | Provide trainings on team development and effective teamwork | - Training on group dynamics, communication, negotiation skills, conflict resolution | [10] |

| **Nr.** | **Dimension** | **Description** | **Sources** |
| --- | --- | --- | --- |
| **9** | **Performance monitoring** | | [1, 6, 8, 10, 14, 16] |
| **9.1** | **Subtheme: Patient** | | [1, 8, 14, 16] |
|  | Instill robust monitoring mechanisms to capture feedback, comments, concerns, compliments, and complaints | - For example, satisfaction surveys, complaints officer/ office - Address concerns, implement suggestions for improvement - Complaints must not lead to disadvantages in the provision of care - Involve patient representatives in performance monitoring | [1, 8, 14, 16, 21]  Survey |
|  | Collect Patient Reported Experience Measures |  | [1, 21]  Survey |
|  | Collect Patient Reported Outcome Measures |  | [1]  Survey |
|  | Collect Patient Reported Adverse Outcomes | - New or worsening symptoms, unanticipated visits to health care facilities | [1] |
| **9.2** | **Subtheme: Caregiver and team** | | [1, 8, 10, 14, 16] |
|  | Give feedback of patient experiences to caregivers |  | [14, 16] |
|  | Support and develop staff to receive feedback in a positive manner |  | [16] |
|  | Collect caregiver experience |  | [1, 8] |
|  | Targeted monitoring and evaluation of individual and team performance for continuous quality improvement |  | [10] |
| **9.3** | **Subtheme: Clinical** | | [6, 8, 14] |
|  | Perform benchmarking |  | [14] |
|  | Perform performance measurement | - Successful implementation of care plan, evidence that the person’s goals are being met, evidence that efforts are being made to minimize difficulties during transitions between healthcare providers and across care settings | [6] |
|  | Perform clinical outcome measurement |  | [8] |

| **Nr.** | **Dimension** | **Description** | **Sources** |
| --- | --- | --- | --- |
| **10** | **Utilizing the potentials of digital health** | | [1, 6, 14] |
| **10.1** | **Subtheme: Structural** | | [1, 6, 14] |
|  | Develop and integrate scalable structures to support health information technology |  | [1] |
|  | Ensure that information technology supports clinicians before, during and after encounters |  | [14] |
|  | Use electronic health records |  | [1, 6]  Survey |
|  | Provide an e-health adoption support | - For clinicians | [1] |
| **10.2** | **Subtheme: Patient-related** | | [1, 14] |
|  | Enable digital visits, as well as in-office visits | - Reimbursement structures should support this | [14] |
|  | Ensure that information technology supports patients before, during and after encounters |  | [14] |
|  | Provide an e-health adoption support for patients (and staff) | - For patients - Ensure a low entry threshold - Enable additional functions for experienced patients | [1] |
|  | Provide patients with self-management tools and information through information technology |  | [14] |
|  | Ensure information technology dynamically tracks patients’ preferences, values, and needs | - For example, “what I would like to be called”. | [14] |
|  | Provide an e-health platform/ smartphone app/ patient portal to give patients access to information, education, their medical record | - Furthermore: contact possibility to clinician, navigation, check symptoms, view wait times for laboratory and pharmacy service | [1, 14]  Survey |
|  | Provide connected patient pathways as an IT-tool including individualized and global information for the patient about his or her disease |  | Survey |

# Works Cited

1. (23) Santana MJ, Manalili K, Jolley RJ, Zelinsky S, Quan H, Lu M. How to practice person-centred care: A conceptual framework. Health Expect. 2018;21:429–40.

2. (29) Byrne A-L, Baldwin A, Harvey C. Whose centre is it anyway? Defining person-centred care in nursing: An integrative review. PLoS One. 2020;15:e0229923.

3. (28) The British Columbia Patient-Centered Care Framework. British Columbia Ministry of Health; 2015.

4. (35) Scholl I, Zill JM, Härter M, Dirmaier J. An integrative model of patient-centeredness - a systematic review and concept analysis. PLoS One. 2014;9:e107828.

5. (32) Olson AW, Stratton TP, Isetts BJ, Vaidyanathan R, C Van Hooser J, Schommer JC. Seeing the Elephant: A Systematic Scoping Review and Comparison of Patient-Centeredness Conceptualizations from Three Seminal Perspectives. J Multidiscip Healthc. 2021;14:973–86.

6. (30) American Geriatrics Society Expert Panel on Person-Centered Care. Person-Centered Care: A Definition and Essential Elements. J Am Geriatr Soc. 2016;64:15–8.

7. (34) Robinson JH, Callister LC, Berry JA, Dearing KA. Patient-centered care and adherence: definitions and applications to improve outcomes. J Am Acad Nurse Pract. 2008;20:600–7.

8. (27) Brickley B, Sladdin I, Williams LT, Morgan M, Ross A, Trigger K, et al. A new model of patient-centred care for general practitioners: results of an integrative review. Fam Pract. 2020;37:154–72.

9. (31) Holmström I, Röing M. The relation between patient-centeredness and patient empowerment: a discussion on concepts. Patient Educ Couns. 2010;79:167–72.

10. (24) People-centred health care: a policy framework. World Health Organization; 2013.

11. (10) Mitchell K-AR, Brassil KJ, Rodriguez SA, Tsai E, Fujimoto K, Krause KJ, et al. Operationalizing patient-centered cancer care: A systematic review and synthesis of the qualitative literature on cancer patients’ needs, values, and preferences. Psychooncology. 2020;29:1723–33.

12. (33) Ouwens M, Hermens R, Hulscher M, Vonk-Okhuijsen S, Tjan-Heijnen V, Termeer R, et al. Development of indicators for patient-centred cancer care. Support Care Cancer. 2010;18:121–30.

13. (40) Castro EM, Van Regenmortel T, Vanhaecht K, Sermeus W, Van Hecke A. Patient empowerment, patient participation and patient-centeredness in hospital care: A concept analysis based on a literature review. Patient Educ Couns. 2016;99:1923–39.

14. (36) Greene SM, Tuzzio L, Cherkin D. A Framework for Making Patient-Centered Care Front and Center. Perm J. 2012;16:49–53.

15. (37) Zucca A, Sanson-Fisher R, Waller A, Carey M. Patient-centred care: making cancer treatment centres accountable. Support Care Cancer. 2014;22:1989–97.

16. (38) Symingtom D. Person Centred Health and Care Framework 2018-2021. NHS Eileanan Siar Western Isles; 2017.

17. (39) Pel E, Engelberts I, Schermer M. Diversity of interpretations of the concept “patient-centered care for breast cancer patients”; a scoping review of current literature. J Eval Clin Pract. 2021. https://doi.org/10.1111/jep.13584.

18. (19) Langberg EM, Dyhr L, Davidsen AS. Development of the concept of patient-centredness - A systematic review. Patient Educ Couns. 2019;102:1228–36.

19. (20) Mead N, Bower P. Patient-centredness: a conceptual framework and review of the empirical literature. Soc Sci Med. 2000;51:1087–110.

20. (please add – X) Louw JM, Marcus TS, Hugo JFM. Patient- or person-centred practice in medicine? - A review of concepts. Afr J Prim Health Care Fam Med. 2017;9:e1–7.

21. (25) Oberst S, van Harten W, Saeter G, de Paoli P, Nagy P, Burrion J, Lovey J, Philip T. 100 European core quality standards for cancer care and research centres. 2020. 100 European core quality standards for cancer care and research centres [accessed 2025-05-29]

22. (17) Giusti A, Nkhoma K, Petrus R, Petersen I, Gwyther L, Farrant L, et al. The empirical evidence underpinning the concept and practice of person-centred care for serious illness: a systematic review. BMJ Glob Health. 2020;5:e003330.

23. (42) Evans JM, Matheson G, Buchman S, MacKinnon M, Meertens E, Ross J, et al. Integrating cancer care beyond the hospital and across the cancer pathway: a patient-centred approach. Healthc Q. 2015;17 Spec No:28–32.

1. Caregiver does not only relate to physicians and nurses but to all staff involved in the care of a patient, for example, including organizational support that may be offered by a case manager or navigation assistance. [↑](#footnote-ref-1)
